# GM1 ganglioside-independent intoxication by Cholera toxin

**DOI:** 10.1371/journal.ppat.1006862

**Published:** 2018-02-12

**Authors:** Jakob Cervin, Amberlyn M. Wands, Anna Casselbrant, Han Wu, Soumya Krishnamurthy, Aleksander Cvjetkovic, Johanna Estelius, Benjamin Dedic, Anirudh Sethi, Kerri-Lee Wallom, Rebecca Riise, Malin Bäckström, Ville Wallenius, Frances M. Platt, Michael Lebens, Susann Teneberg, Lars Fändriks, Jennifer J. Kohler, Ulf Yrlid

**Affiliations:** 1 Department of Microbiology and Immunology, Institute of Biomedicine, University of Gothenburg, Gothenburg, Sweden; 2 Department of Biochemistry, University of Texas Southwestern Medical Center, Dallas, Texas, United States; 3 Department of Gastrosurgical Research and Education, Institute of Clinical Sciences, Sahlgrenska Academy, University of Gothenburg, Gothenburg, Sweden; 4 Department of Biochemistry and Cell Biology, Institute of Biomedicine, Sahlgrenska Academy, University of Gothenburg, Gothenburg, Sweden; 5 Department of Pharmacology, University of Oxford, Oxford, United Kingdom; 6 Sahlgrenska Cancer Center, University of Gothenburg, Gothenburg, Sweden; 7 Mammalian Protein Expression Core Facility, University of Gothenburg, Gothenburg, Sweden; McMaster University, CANADA

## Abstract

Cholera toxin (CT) enters and intoxicates host cells after binding cell surface receptors via its B subunit (CTB). We have recently shown that in addition to the previously described binding partner ganglioside GM1, CTB binds to fucosylated proteins. Using flow cytometric analysis of primary human jejunal epithelial cells and granulocytes, we now show that CTB binding correlates with expression of the fucosylated Lewis X (Le^X^) glycan. This binding is competitively blocked by fucosylated oligosaccharides and fucose-binding lectins. CTB binds the Le^X^ glycan *in vitro* when this moiety is linked to proteins but not to ceramides, and this binding can be blocked by mAb to Le^X^. Inhibition of glycosphingolipid synthesis or sialylation in GM1-deficient C6 rat glioma cells results in sensitization to CT-mediated intoxication. Finally, CT gavage produces an intact diarrheal response in knockout mice lacking GM1 even after additional reduction of glycosphingolipids. Hence our results show that CT can induce toxicity in the absence of GM1 and support a role for host glycoproteins in CT intoxication. These findings open up new avenues for therapies to block CT action and for design of detoxified enterotoxin-based adjuvants.

## Author summary

The disease cholera, caused by cholera toxin produced by *Vibrio cholerae*, is responsible for over 100 000 deaths every year. When taken up by cells in the intestine, the toxin causes diarrhea, and the ganglioside GM1 (a glycolipid) has long been considered the main receptor for cholera toxin. We here present data showing that cholera toxin binds to fucose within the trisaccharide blood group antigen Lewis X. Lewis X is expressed on human granulocytes as well as intestinal cells and correlates with cholera toxin binding. Furthermore, cell lines deficient in GM1 expression and mice lacking GM1 treated to further reduce glycolipid synthesis are intoxicated by cholera toxin. This indicates an active role of glycoproteins, and our results thereby provide new insights on how cholera toxin is internalized into cells in the human intestine. This knowledge can be used to design novel drug candidate inhibitors of cholera toxin. Another way to utilize our findings is to modify cholera toxin to be less toxic so that it can be used as an adjuvant in vaccines.

## Introduction

The disease cholera is well known for the acute diarrhea that if left untreated can be lethal within hours. The World Health Organization (WHO) estimates that over 100 000 people die every year from cholera infection and children under five are the most susceptible. The vast majority of patients treated with isotonic intravenous and oral fluids (Ringer´s solution) survive while in untreated cases the mortality rate can reach 50% [1]. The disease is caused by the gram-negative bacterium *Vibrio cholerae* that is naturally present in the water environment in some tropical areas such as the Ganges delta and most of sub-Saharan Africa. A recent development is the spreading of *V*. *cholerae* to Haiti where cholera is now an endemic disease [2].

There are several serotypes of *V*. *cholerae* but only O1 and O139 cause endemic cholera and produce cholera toxin (CT), the main causative agent of the extreme intestinal fluid secretion characteristic of cholera patients [3]. CT is composed of one A-subunit (CTA) with a non-covalently associated pentameric ring of B-subunits (CTB). The B-subunits bind to the intestinal cell surface and facilitate retrograde transport of CT via the endoplasmic reticulum into the cytoplasm. Before entering the cytoplasm, CTA is detached from CTB and is then further cleaved to CTA1. CTA1 then catalyzes ADP-ribosylation of Gs-α which in turn constitutively activates adenylate cyclase leading to elevated intracellular levels of cAMP. cAMP acts as a message to activate protein kinase A that phosphorylates the cystic fibrosis transmembrane conductance regulator (CFTR). The ion channel CFTR allows secretion of chloride ions into the intestinal lumen and due to osmotic pressure, water will follow leading to an acute watery diarrhea [4–6].

The ganglioside GM1 has long been considered the main functional receptor for CT. This idea is supported by the fact that GM1 binds with high affinity to both CTB and the closely related heat-labile toxin B-subunit from *E*. *coli* (LTB) [7]. Furthermore, addition of exogenous ganglioside GM1 to rabbit ileum significantly increases the response to CT, a finding that has been reproduced in murine and human cell lines [8–10]. Together, these data strongly suggest that intestinal GM1 acts as a functional receptor for CT. Extensive work elucidating the pathway CT takes from cell surface to cytoplasm shows that CT binds to detergent-resistant parts of the cell membrane on various cell types, consistent with the idea that GM1 is enriched in these regions [11,12]. Indeed, CTB is commonly used as a marker for lipid rafts as well as GM1 [11,13]. CTB is also known to bind with varying affinities to oligosaccharide (os) portions of GM1-related glycosphingolipids (GSLs) such as Fuc-GM1, GM2, GD1a, GM3, GT1b, GD1b and asialo-GM1 [13,14]. However, the level of GM1 in the human intestine is very low compared to other GSLs, raising the question as to whether the amount of GM1 is sufficient to create the lethal diarrheal response that is seen in some individuals [8,15].

Already almost forty years ago, glycoproteins that bound CT were identified in lysates of rat intestines [16]. Subsequent studies made in other species gave support to the existence of glycoproteins binding to CT(B) [17–19]. Recently, we reported that CTB binds to fucosylated glycoproteins on primary human colonic cells and cell lines [20]. In the course of these studies, we attempted to measure the contribution of GM1 as a cell surface receptor for CTB, but it proved to be undetectable in the colonic cell line T84 that is commonly used to mimic the human intestinal response to CT [3,6,20]. While our experiments indicated that fucose played a key role in binding, our data did not provide insight into the nature of the fucosylated glycans recognized by CTB. Indications of which fucosylated glycan moieties could bind CTB were recently revealed by structural studies showing binding of histo blood group antigens (HBGAs) to CTB and LTB [21–23]. HBGAs are present at high concentrations in the human intestinal epithelium. Epidemiological studies have also shown that although people with blood group O appear to have a lower risk of getting infected with *V*. *cholerae*, those who do get infected have an elevated risk of developing more severe symptoms [24–26]. These findings suggest that intestinal glycan moieties other than GM1 may be able to bind CT, and that fucosylated HBGAs are of particular interest.

In this study we have investigated the binding of CTB to freshly isolated human primary cells. We identify that CTB binds to the blood group antigen Lewis^X^ (Le^X^), and that this binding is fucose-dependent both *in vitro* and *ex vivo*. Furthermore, we show that CT can intoxicate cells as well as mice lacking GM1 even after additional inhibition of GM1-related glycosphingolipid (GSL) biosynthesis.

## Results

### CTB-binding fucosylated glycoconjugates are expressed at high levels on the surface of human granulocytes

**Fig 1:**
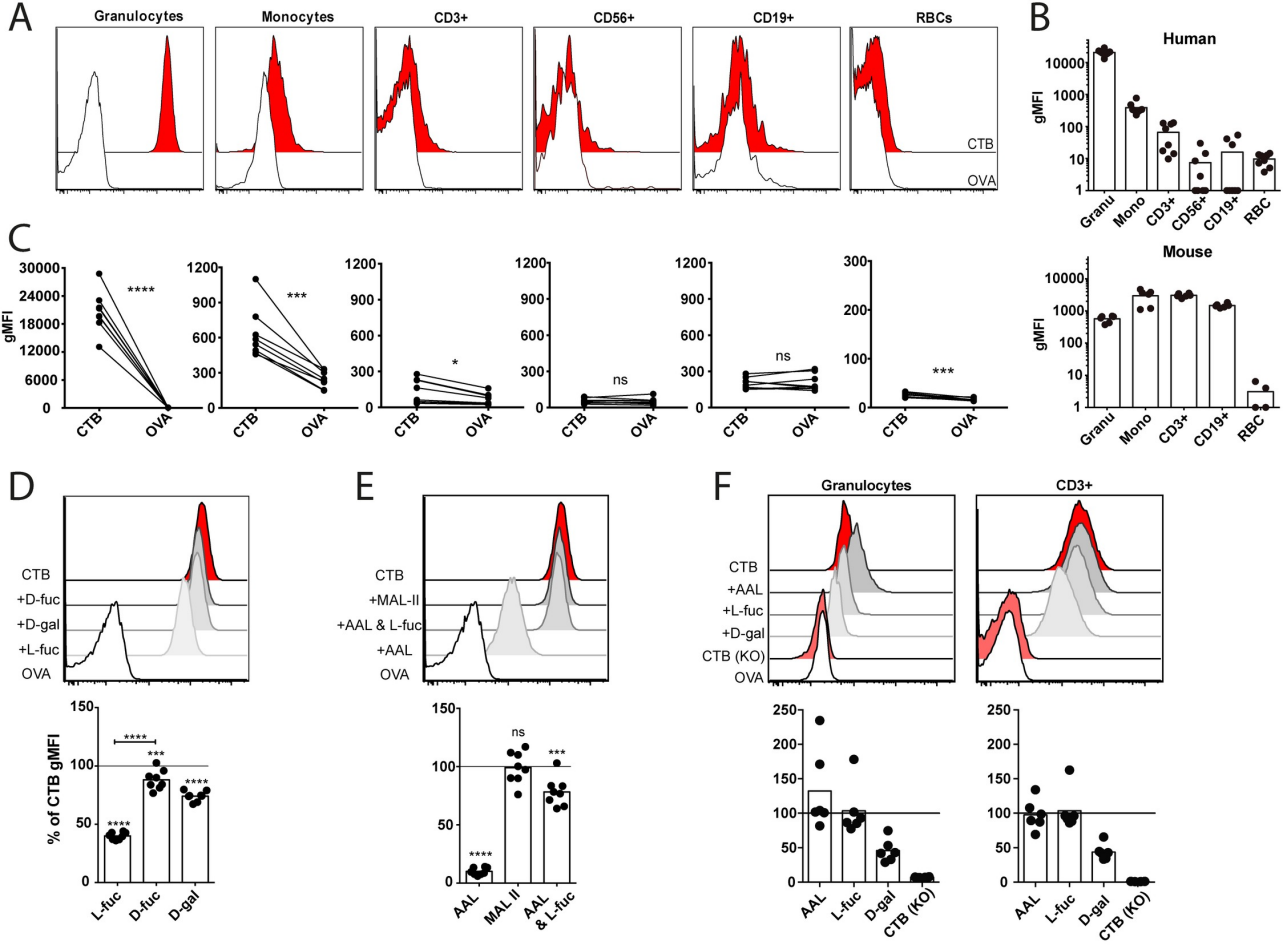
CTB binds to fucosylated structures expressed on human granulocytes. **(A)** Representative histograms from flow cytometry analysis of CTB- (red) and OVA- (white) binding to cell types in human peripheral blood. **(B)** Bar graphs show the geometric mean fluorescent index (gMFI) of CTB binding (with OVA gMFI subtracted) to human whole blood (n = 8) and murine splenocytes (n = 4–6). Each dot represents one donor/mouse. **(C)** gMFI of CTB and OVA binding to the different cell types from human whole blood. Each pair of dots represents one donor. **(D-E)** Histogram and bar graph (n = 8) showing blocking of CTB binding to human granulocytes by pre-treating CTB with **(D)** L-, D-fucose and D-galactose or **(E)** with the lectins AAL or MAL-II. **(F)** Histograms and bar graphs (n = 6) showing the blocking of CTB binding to murine (wt or KO) cells by either pre-treating the cells with lectins or pre-treating CTB with sugars. For panel **(C)** significance was calculated using paired t-test and for **(D-E)** one-way-ANOVA was used with Tukey correction (*** = p<0,005, **** = p<0,0001).

Several studies have suggested a link between ABO blood group type and risk of severe symptoms of cholera disease [19,27,28]. A recent study reporting crystal structures of CTB complexes with HBGAs has also shown that the determinant of blood group O (i.e. the H antigen) can bind CTB in two orientations [21]. We therefore stained peripheral blood cells from healthy donors of blood group A, B, AB or O with CTB-biotin, followed by fluorescently-labeled streptavidin and performed flow cytometric analysis. FSC/SSC characteristics and a panel of mAbs were used to identify red blood cells and subsets of leukocytes. We found that red blood cells (RBCs) exhibited very low but significant levels of staining with CTB. However, no difference in staining pattern of RBC was observed between blood groups A, B, AB or O (S1 Fig). However, granulocytes from all blood group donors stained most brightly with CTB; roughly two logs higher than monocytes and T cells that also showed significant staining above the background observed with biotinylated Ovalbumin (OVA) used as negative control (Fig 1A–1C). In contrast, B cells and NK cells did not stain with CTB (Fig 1A–1C). No significant differences in staining patterns of monocytes, granulocytes or T cells were observed between donors of blood group A, B, AB or O (S1 Fig).

We have recently shown that in addition to GM1, CTB binds to fucosylated glycoproteins present on the cell surface [20]. Therefore competitive inhibition analysis of CTB-biotin binding to peripheral white blood cells by monosaccharides was performed. Our previous study had determined that galactose efficiently blocked binding to Jurkat cells that express high levels of GM1, while fucose was the best blocker of CTB binding to colonic epithelial cell lines [20]. We observed that L-fucose efficiently inhibited the binding of CTB-biotin to granulocytes, whereas D-fucose and D-galactose were much less potent (Fig 1D). Binding of CTB-biotin to monocytes was inhibited by monosaccharides, but on these cells the most efficient block was obtained with D-galactose followed by D- and L-fucose (S1 Fig). The fucose-recognizing *Aleuria aurantia* lectin (AAL) also efficiently blocked binding of CTB to granulocytes (Fig 1E). Pre-treating AAL with L-fucose counteracted this blocking effect, underscoring that fucosylated structures on the surface of granulocytes are recognized by CTB. In addition, neither the sialic acid-binding *Maackia amurensis* lectin II (MAL-II) lectin (Fig 1E) nor the galactose-binding Peanut Agglutinin (PNA) (S1 Fig) blocked the binding of CTB to granulocytes.

In contrast to human leukocytes, murine leukocytes regardless of subtype bound CTB-biotin efficiently and to comparable levels (Fig 1B). This binding was not affected by AAL or L-fucose but inhibited by D-galactose (Fig 1F). PNA did not block CTB binding to murine lymphocytes (S1E Fig). In mice deficient in β4GalNAc-transferase (B4galnt1), which have no GM1 and drastically reduced levels of GM1-related GSLs (such as Fuc-GM1, GM2, GD1a, GT1b, GD1b and asialo-GM1) [29], binding of CTB to leukocytes could not be detected (Fig 1F). This suggests that the observed binding of CTB to murine leukocytes is not dependent on fucosylated structures, but rather on GM1 and the GM1-related GSLs. Collectively, these experiments show that CTB binds to fucosylated structures that are abundantly expressed on the surface of human granulocytes, but that CTB binding to mouse granulocytes lacks a strong fucose dependence.

### CTB binds to Le^X^-carrying proteins but not to Le^X^-carrying ceramide

**Fig 2:**
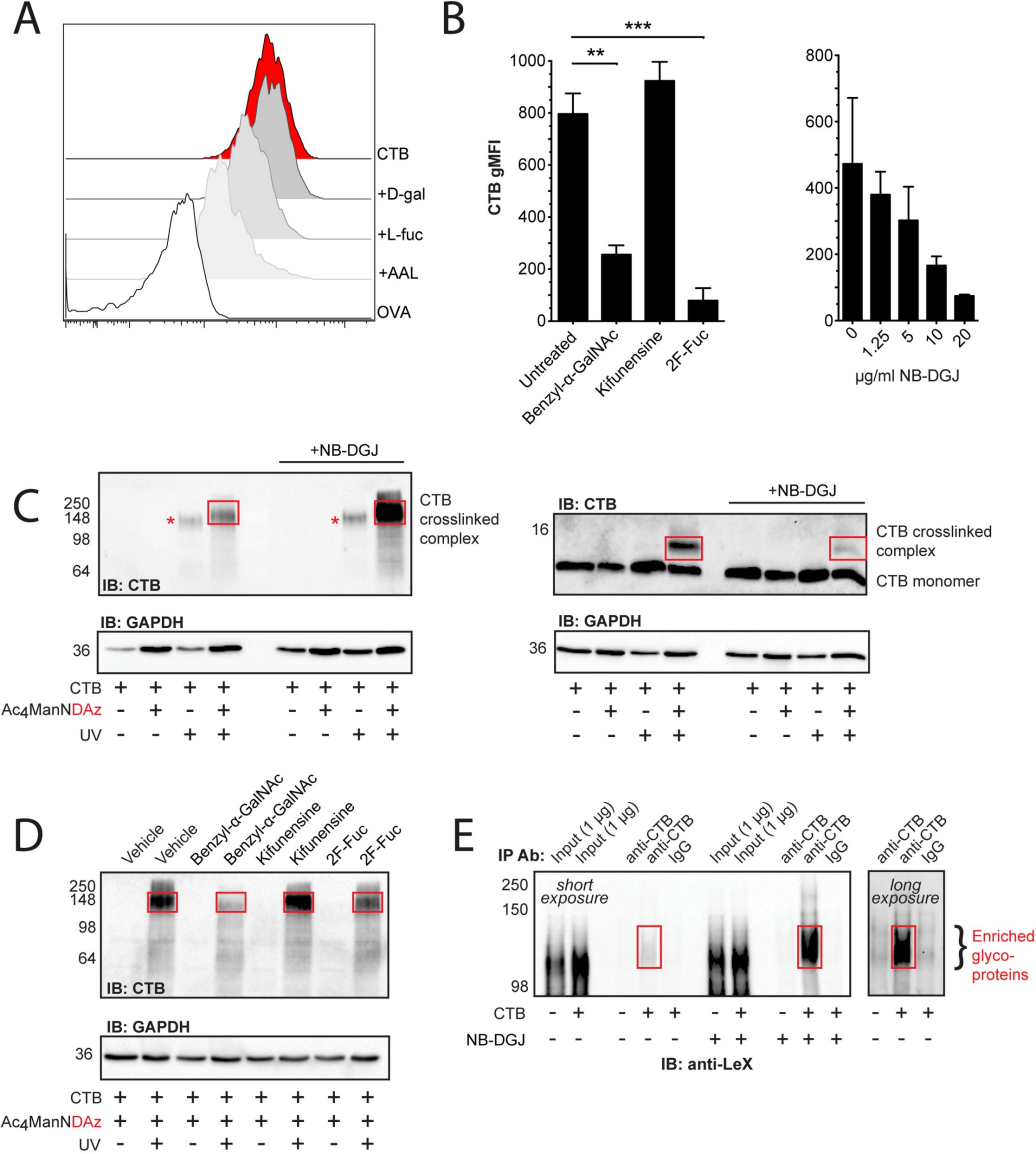
CTB binds to Le^X^-carrying proteins in HL-60 cells. **(A)** Histogram from flow cytometry analysis of CTB-binding to HL-60 cells following pre-treatment of the cells with AAL (10 μg/ml) or pre-treatment of CTB with sugars (50 mM). **(B)** gMFI of CTB binding to HL-60 cells cultured with the indicated inhibitors (*** = p<0.001 and ** = p<0.01). **(C-D)** Western blot using anti-CTB of HL-60 cells co-cultured with **(C)** (NB-DGJ) or **(D)** (benzyl-α-GalNAc, kifunensine, or 2F-Fuc) and the precursor sugar Ac4ManNDAz to enable UV-crosslinking between CTB and glycosylated structures. Representative of two independent experiments. **(E)** Western blot using anti-Le^X^ of HL-60 cells after incubation with CTB, lysis and immunoprecipitation with anti-CTB. One representative out of two independent experiments is shown.

To biochemically assess the CTB binding structure in human myeloid cells we used the promyelocytic leukemia cell line HL-60 that is often used as a model for human granulocytes. HL-60 cells efficiently bound CTB-biotin and this interaction could be inhibited by AAL and L-fucose (Fig 2A). We next cultured undifferentiated HL-60 cells in the presence of inhibitors of protein glycosylation: benzyl-α*-*GalNAc (decoy substrate that interferes with GalNAc-type O-linked protein glycosylation), kifunensine (inhibits maturation of N-linked protein glycosylation) or 2F-Fuc (metabolic inhibitor of fucosylation) [20,30,31]. The activity of the inhibitors was confirmed by lectin binding experiments (S2 Fig). Inhibition of O-linked glycosylation and fucosylation each reduced the binding of CTB, while blocking N-linked glycosylation had no effect (Fig 2B). Growing the cells with NB-DGJ, a compound that interferes with glucosylation of ceramide, an early step in GSL biosynthesis [32] also inhibited (in a dose-dependent manner) the binding of CTB-biotin to HL-60 cells (Fig 2B). To probe for the identity of the structure binding to CTB, we cultured HL-60 cells in the presence of a cell-permeable precursor sugar (Ac4ManNDAz) that can be metabolized to a photo-crosslinking sialic acid analog (SiaDAz) and incorporated into glycoconjugates in place of natural sialic acids [33]. By adding CTB to Jurkat and T84 cells grown with Ac4ManNDAz, we have recently shown that CTB crosslinks glycolipids and glycoproteins respectively in these cell lines [20]. In HL-60 cells, crosslinking with CTB resulted in two bands revealed by anti-CTB immunoblotting (Fig 2C). The molecular weight of the higher band (left panel) is consistent with crosslinking to glycoproteins, while the lower band was consistent with crosslinking to a glycosphingolipid (right panel). Growing the HL-60 cells with the glycolipid inhibitor NB-DGJ increased the intensity of the high molecular weight band and decreased the intensity of the low molecular weight band (Fig 2C), suggesting that removal of the CTB glycolipid binding partners results in enhanced binding of the toxin to its glycoprotein binding partners. Furthermore, consistent with the cell surface binding data (Fig 2B), the intensity of the high molecular weight glycoprotein band was decreased when HL-60 cells were grown with the inhibitor of O-linked glycosylation (benzyl-α-GalNAc) or of fucosylation (2F-Fuc), but not that of the inhibitor of N-linked glycosylation (kifunensine) (Fig 2D). These results imply that CTB binds to two classes of molecules on the surface of HL-60 cells: glycosphingolipids and fucosylated glycoproteins.

Lewis^X^ (Le^X^ or CD15) is a fucosylated HBGA abundantly expressed by granulocytes and HL-60 cells, and we could readily detect this in flow cytometry using an anti-Le^X^ antibody (S2 Fig). Le^X^-like human milk oligosaccharides (HMOs) have previously been shown to bind CTB and LTB [34]. A recent co-crystallization study has shown that the HBGA Lewis^y^ (Le^y^) can bind CTB [21]. The Le^X^ trisaccharide is similar to the Le^y^ tetrasaccharide (Fucα2Galβ4[Fucα3]GlcNAc), but lacks the terminal α1-2-linked fucose residue. Since the α1-2-linked fucose shows no direct contacts to CTB in the co-crystal structure of CTB and Le^y^, we hypothesized that Le^X^ displayed on glycoproteins or glycolipids could bind CTB. CTB-bound proteins within HL-60 cell lysates were therefore immunoprecipitated with an anti-CTB antibody and immunoblotted with anti-Le^X^. This revealed a high molecular weight band of similar size to that detected following crosslinking with CTB and immunoblotting with anti-CTB (Fig 2C and 2E). As expected the covalent crosslinking resulted in a slight increase in molecular weight of this band (Fig 2C compared to 2E). Growing the cells in NB-DGJ intensified the bands detected by the anti-Le^X^ antibody, suggesting that the bands represent glycoproteins rather than glycolipids and that these glycoproteins are modified with Le^X^ and also bind CTB (Fig 2E).

**Fig 3:**
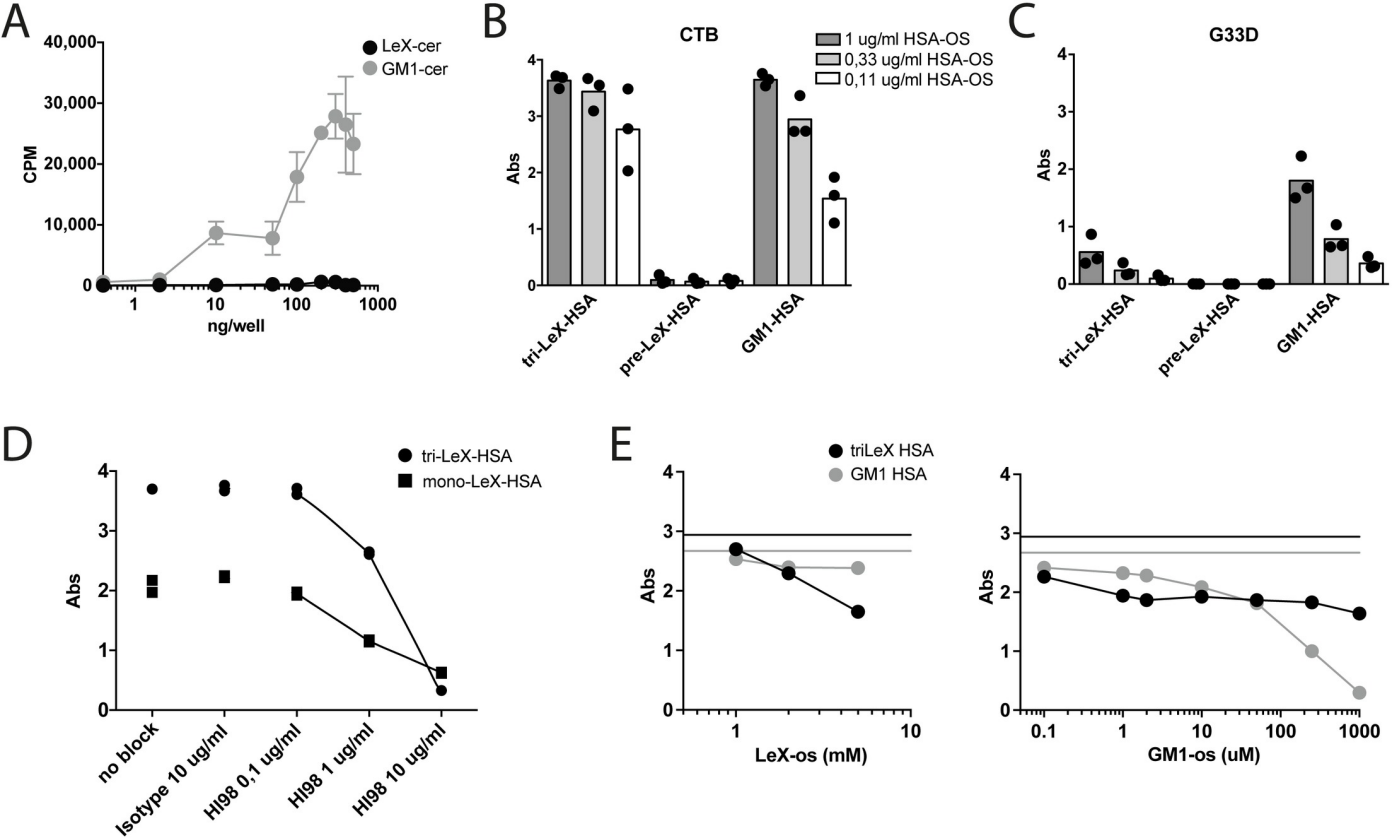
CTB binds to Le^X^ linked to proteins but not ceramide. **(A)** GM1- or Le^X^-os (oligosaccharide) linked to ceramide and immobilized to wells were detected with ^125^I labeled CTB. Relative binding is displayed as counts per minute (CPM). **(B-C)** ELISA with titrated amounts of os-linked to HSA, immobilized to wells and detected with **(B)** CTB-HRP and **(C)** G33D-HRP. Graph shows absorbance values from three independent experiments. **(D)** ELISA with os-linked to HSA, immobilized in wells and then blocked with indicated concentrations of anti-Le^X^ antibody HI98 or isotype control (IgM), and detected with CTB-HRP. Graph shows absorbance values from one representative out of two independent experiments. **(E)** ELISA with os-linked to HSA, immobilized in wells and detected with CTB-HRP in the presence of increasing blocking concentrations of tri-Le^X^-os and GM1-os. Graphs show absorbance values from one representative out of two independent experiments.

To directly assess if CTB could interact with lipid-linked Le^X^, a radioimmuno assay (RIA) was performed, using CTB labeled with ^125^I and purified glycosphingolipids covalently modified with either Le^X^ or GM1. No binding of ^125^I-labelled CTB was observed when Le^X^ was linked to a ceramide, while a clear dose-dependent binding of CTB to GM1 oligosaccharide linked to ceramide was detected (Fig 3A). In contrast, when Le^X^ (Galβ4(Fucα3)GlcNAc), tri-Le^X^ (Galβ4(Fucα3)GlcNAcβ3Galβ4(Fucα3)GlcNAcβ3Galβ4(Fucα3)GlcNAc) or GM1 (Galβ3GalNAcβ4(Neu5Ac2-3)Galβ4Glc) were linked to human serum albumin (HSA) (using an APE-linker) and coated to plates, CTB-HRP efficiently detected all glycoproteins (Fig 3B and 3D). The binding to Le^X^ was completely dependent on the fucose as no binding to Galβ4GlcNAc (LacNAc or CD75—here called pre-Le^X^) was detected by CTB-HRP (Fig 3B). The fucose-binding lectin AAL did not bind to HSA-GM1 but bound efficiently to HSA-Le^X^ and could thereby inhibit subsequent binding of CTB to HSA-Le^X^ (S3 Fig). The galactose-binding PNA did not bind to HSA-Le^X^ but bound HSA-GM1. This binding did not, however, block the subsequent binding of CTB to HSA-GM1 (S3 Fig). The binding of CTB to mono- and tri-Le^X^-HSA was dose-dependently blocked by the Le^X^-specific mAb (HI98) further corroborating the specificity of the interaction between CTB and Le^X^ (Fig 3D). Collectively, these results show that CTB binds to Le^X^ when this moiety is displayed on proteins but not ceramide, and that this binding is dependent on the α1,3-linked fucose and can be blocked by mAb to Le^X^.

### CTB binds Le^X^ but binding of the G33D mutant is greatly reduced

The site used by CTB to bind to the GM1 glycan has been identified by crystallography and confirmed by mutagenesis. While most contacts with GM1 are made by amino acids from a single CTB monomer, the Gly33 residue of an adjacent monomer also contributes to solvent-mediated contact with GM1. A point-mutated variant of CTB (G33D) in which aa 33 is exchanged from glycine to aspartic acid has been shown to exhibit greatly reduced binding to GM1 [35,36]. However a recent crystal structure suggests that blood group determinants can bind CTB using a site on the lateral surface of CTB approximately 18 Å from Gly33 [21]. As expected, G33D-HRP showed a decreased binding to GM1-HSA as compared to CTB-HRP (Fig 3C). But surprisingly, using G33D-HRP to detect plate-bound tri-Le^X^-HSA also showed a greatly reduced binding compared to CTB-HRP (Fig 3C). Thus, a mutation in the GM1 binding pocket affects recognition of Le^X^ by CTB. To further assess this, plate-bound GM1-HSA or tri-Le^X^-HSA were tested for binding of CTB-HRP that had been pre-incubated with increasing concentrations of GM1-os or Le^X^-os. GM1-os blocked the binding of CTB to GM1-HSA in a dose-dependent manner and less potently (reaching saturation at around 1 μM) to tri-Le^X^-HSA (Fig 3E). In contrast, Le^X^-os blocked the binding of CTB to tri-Le^X^-HSA but not GM1-HSA (Fig 3E). The concentration required to obtain blocking of CTB binding to tri-Le^X^-HSA was roughly a 1000-fold higher with Le^X^-os compared to GM1-os (Fig 3E). The decreased binding of the G33D mutant to tri-Le^X^-HSA as well as the partial competition between GM1-os and Le^X^-os suggest that GM1 and Le^X^ may be accommodated in the same binding site of CTB, with higher apparent binding affinity for GM1 as compared to Le^X^. Alternative explanations for these results include allosteric communication between the canonical GM1 binding site and the novel fucosylated glycan binding site identified in the CTB-Le^y^ crystal structure, [21] or Le^X^ binding to both sites.

**Fig 4:**
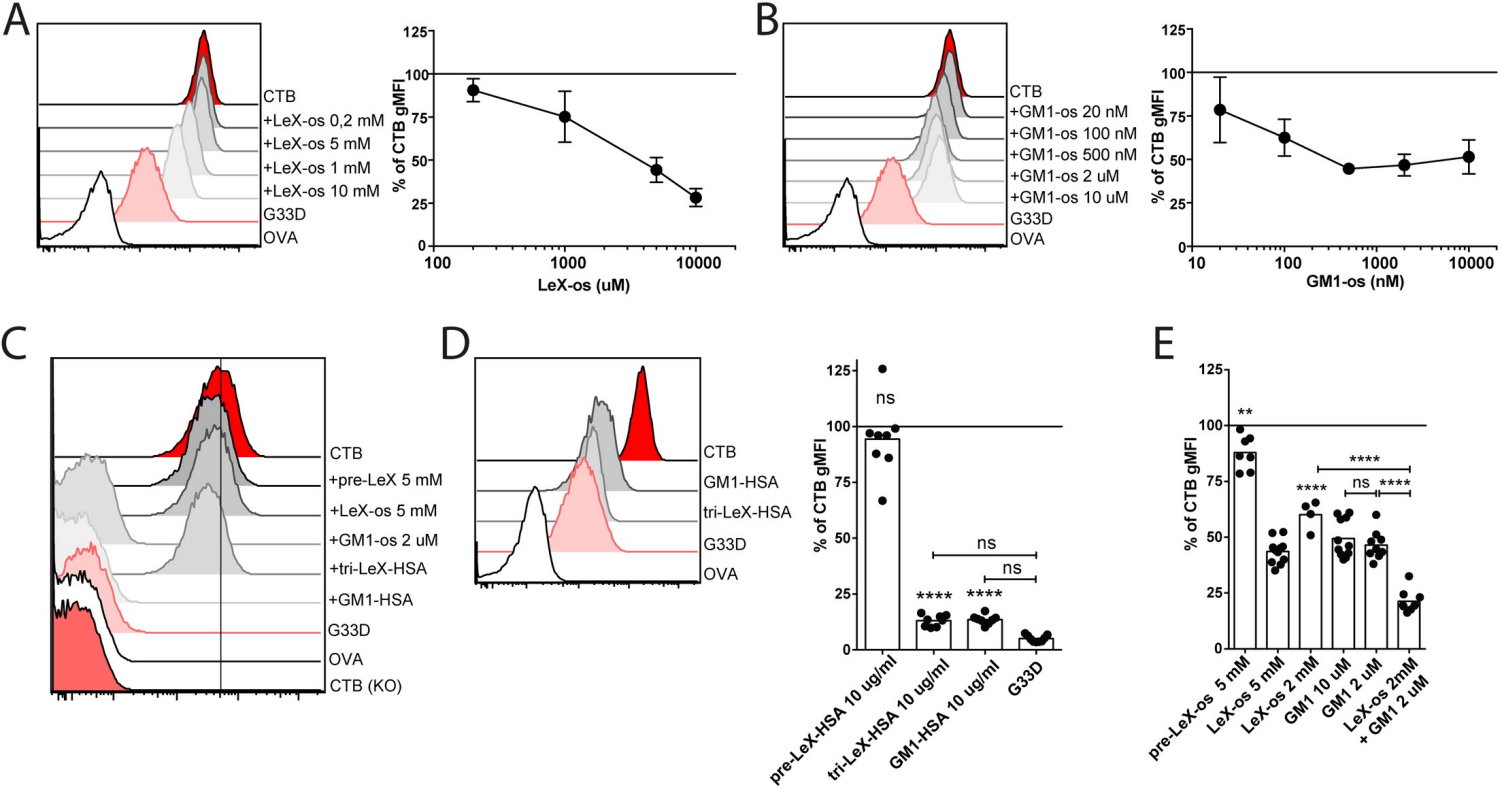
Le^X^ blocks binding of CTB to human granulocytes but not murine leukocytes. **(A-B and D-E)** Histograms from flow cytometry analyses of CTB-, G33D- and OVA-binding to granulocytes in human peripheral blood. CTB was pretreated or not with titrated amounts of **(A)** Le^X^-os, **(B)** GM1-os, **(D)** os-HSA (not titrated) and **(E)** Le^X^-os and GM1-os. Graphs show the percent of gMFI of CTB binding to the cells where 100% represents CTB staining with no blocking oligosaccharide. **(C)** Histograms from flow cytometry analyses of CTB-, G33D- and OVA-binding to CD3+ T cells gated from murine splenocytes. CTB was pretreated or not with the indicated os or os-HSA. **(A-B)** n = 3, **(C)** One representative out of three independent experiments, **(D)** n = 8, **(E)** n = 4–9. Error bars show SD. Each dot represents one donor and significance was calculated using a one-way-ANOVA with Tukey correction compared to CTB without block if not indicated otherwise with bars (**** = p<0,0001, *** = p<0,005, ** = p<0,01).

We next evaluated if pre-incubating CTB with Le^X^-os and GM1-os also could block the binding of CTB to human peripheral blood leukocytes and in particular to granulocytes, where our data had indicated that fucosylated structures are the primary contributors to CTB binding. As observed in the ELISAs, at least a 1000-fold higher concentration of Le^X^-os compared to GM1-os was required to efficiently block the binding of CTB to granulocytes (Fig 4A and 4B). However, and consistent with the ELISA data in Fig 3E, GM1-os did not fully block CTB binding even at high concentrations. In contrast, the binding of CTB to murine lymphocytes, where GM1 is the main contributor to CTB binding, was not blocked by similar doses of Le^X^-os but completely inhibited by GM1-os (Fig 4C). Likewise the binding of CTB to Jurkat cells, expressing high levels of GM1, was not blocked by PNA, AAL or Le^X^-os but by GM1-os (S3 Fig). Addition of tri-Le^x^-HSA, thereby potentially increasing the multivalency and steric hindrance of the interaction, resulted in a partial block of the binding of CTB to GM1 or GM1-related GSLs on the surface of murine leukocytes and Jurkat cells (Fig 4C and S3 Fig). Using tri-Le^X^ or GM1 linked to HSA efficiently blocked the binding of CTB to human granulocytes down to levels obtained with G33D (Fig 4D). Combining an unsaturated level of Le^X^-os with GM1-os at a saturated level (Fig 4A and 4B) significantly reduced the level of CTB-biotin binding to granulocytes (Fig 4E). Collectively, these results show that CTB primarily binds Le^X^ on human granulocytes through the same site that binds GM1 and GM1-related GSLs on murine lymphocytes, but that high affinity binding of GM1-os does not completely block the low affinity binding of CTB to Le^X^-os.

### CTB binds epithelial cells from human jejunum and this capacity is elevated among the cells that express Le^X^

**Fig 5:**
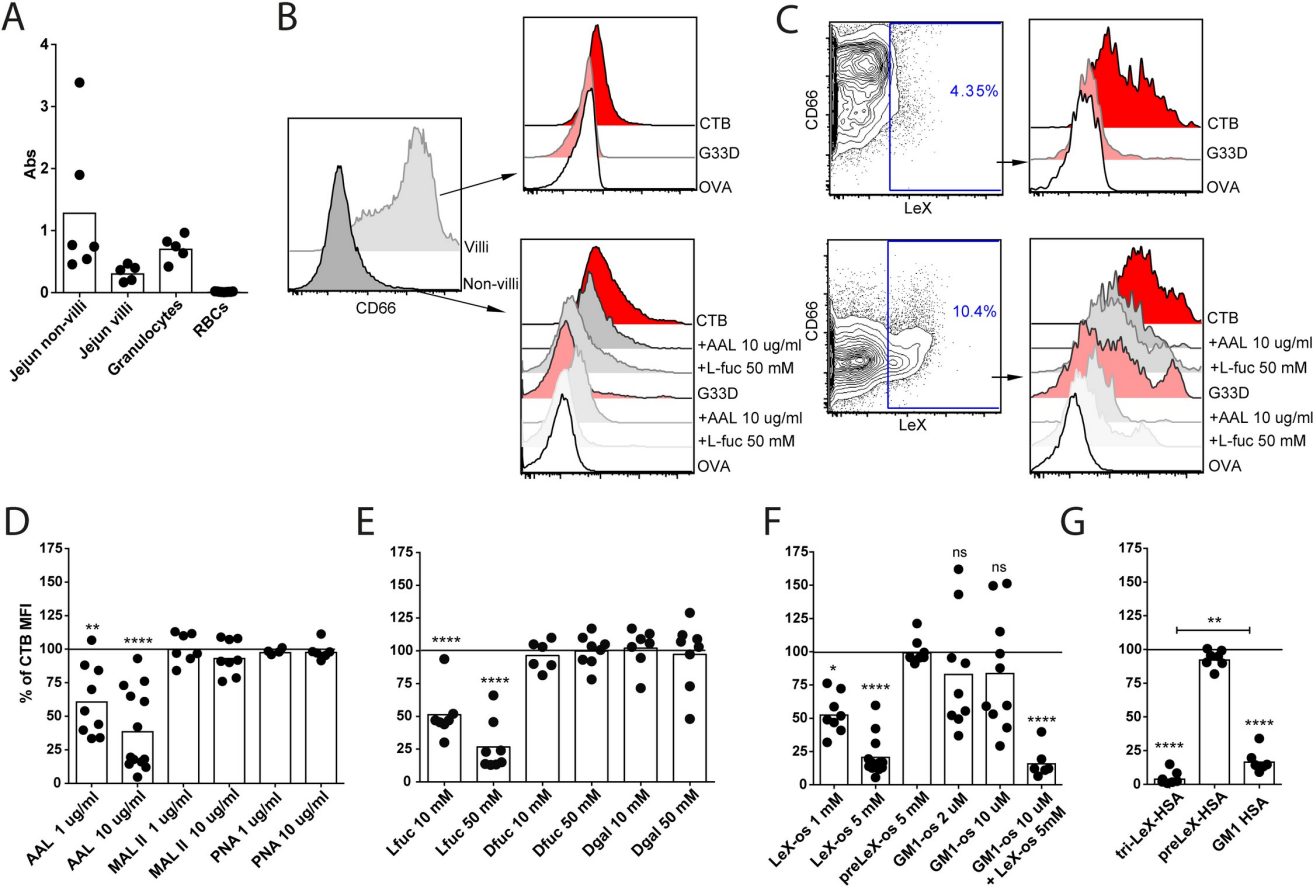
CTB binding to primary human intestinal cells can be blocked by interference with fucosylated structures. **(A)** Bar graph showing relative absorbance values from an ELISA with immobilized anti-Le^X^, and detection with CTB-HRP. Samples as indicated from lysates of isolated human cells (2 μg protein/ml). Each dot represents a human donor (n = 5–8). **(B)** CD66 or **(C)** CD66 and Le^X^ expression by jejunal epithelial cells that were isolated using EDTA medium (villi) or enzymatic degradation after EDTA treatment (non-villi or crypt). Histograms from flow cytometry analyses of CTB-, G33D- and OVA-binding to the differentially enriched epithelial cells. **(B)** EpCAM^+^ cells and **(C)** EpCAM^+^Le^X+^ cells. **(D-G)** Bar graph showing percent of gMFI of CTB binding to jejunal epithelial cells by pretreatment of the cells with **(D)** lectins, **(E)** sugars, **(F)** oligosaccharides and **(G)** HSA-linked oligosaccharides. Graphs show the percent of gMFI of CTB binding to the cells where 100% represents CTB staining with no blocking oligosaccharide. Each dot represents a donor in **(D)** n = 4–12, **(E)** n = 6–8, **(F)** n = 6–12, **(G)** n = 6–7. Significance was calculated using a one-way-ANOVA with Tukey correction compared to CTB without block if not indicated otherwise with bars (**** = p<0,0001, *** = p<0,005, ** = p<0,01 and * = p<0,05).

We have recently shown that CTB binds to glycoproteins on the human colonic cell line T84 [20]. To determine if these CTB-binding glycoproteins are modified with Le^X^, CTB-bound proteins within T84 cell lysates were immunoprecipitated with an anti-CTB antibody and immunoblotted with anti- Le^X^. This analysis revealed several bands of high molecular weight (S4 Fig). The recognized glycoproteins were of similar size to those detected following immunoblots with CTB-HRP of T84 cells [20]. We next wanted to assess if primary human tissues, and in particular jejunum where the majority of fluid secretion following CT intoxication occurs, contained CTB-binding proteins expressing Le^X^. An ELISA with anti-Le^X^ as the capture mAb and CTB-HRP as the detection reagent was therefore used. Le^X^-expressing and CTB-binding proteins were detected in lysates from human granulocytes but not in RBCs, which is in line with our flow cytometric detection of CTB-binding cells in human peripheral blood (Figs 1A and 5A). In addition, lysates of cells from jejunum contained Le^X^-expressing CTB-binding proteins (Fig 5A). Cells collected after an initial incubation with EDTA primarily contain epithelial cells from jejunal villi and the upper part of colonic crypts [37]. Enzymatic digestion of the remaining jejunal tissue enriches for more immature epithelial cells from the crypt and cells from the lamina propria. CD66a has been shown to be expressed at low levels on human immature epithelial cells from the bottom of the crypt and then increase as the epithelial cells mature and move upward along the colonic crypts [37]. The enrichment of epithelial cells from these regions was confirmed by flow cytometric analysis of CD66a expression (Fig 5B). The amount of Le^X^-expressing CTB-binding proteins appeared to be increased in jejunal cells from the crypts compared to jejunal villi (Fig 5A). Similarly, the level of CTB binding to the surface of jejunal epithelial cells from the crypt was increased compared to the villi (Fig 5B). Flow cytometry analysis also showed that a fraction of jejunal epithelial cells expressed Le^X^ (Fig 5C). In addition, this fraction was increased in epithelial cells from the crypt and these cells stained brightly with CTB (Fig 5C). No staining with G33D was detected to epithelial cells from the villi but binding was observed to Le^X^-expressing epithelial cells from the crypt (Fig 5B and 5C). This binding of G33D was blocked by the fucose-binding lectin AAL and L-fucose (Fig 5C). AAL, but not sialic acid-binding MAL-II or galactose-binding PNA, efficiently blocked binding of CTB-biotin to jejunal epithelial cells (Fig 5D).

Pre-incubating CTB with L-fucose but not D-fucose or D-galactose blocked the binding to the jejunal epithelial cells (Fig 5E). Furthermore, Le^X^-os and tri-Le^X^-HSA but not pre-Le^X^-os or pre-Le^X^-HSA inhibited the binding of CTB to epithelial cells (Fig 5F and 5G). The consequence of adding competing GM1 oligosaccharide to CTB differed between human donors as this resulted in either increased or decreased binding of CTB (Fig 5F). However, pre-incubation of CTB with GM1-HSA efficiently blocked the binding of CTB (Fig 5G). Interestingly, in about 30% of the donors, all epithelial cells expressed Le^X^ (S5 Fig). This staining highlighted that the level of CTB binding correlated with level of Le^X^ expression as a diagonal was observed in the contour plot (S5 Fig). This subgroup did not cluster regarding the results obtained with the competitive blocking experiments (S5 Fig). However, as all cells expressed Le^X^, evaluation of blocking CTB binding to epithelial cells by anti-Le^X^ could be performed and showed a dose dependent inhibition of CTB binding with anti-Le^X^ (S5D and S5E Fig). Taken together, these results show that CTB binds to fucosylated structures including Le^X^ on human primary jejunal epithelial cells, and that the level of CTB-binding is increased in Le^X^-expressing jejunal cells and can be blocked with an anti-Le^X^ mAb.

### AAL and PNA efficiently block CT-induced secretion by human jejunal biopsies

**Fig 6:**
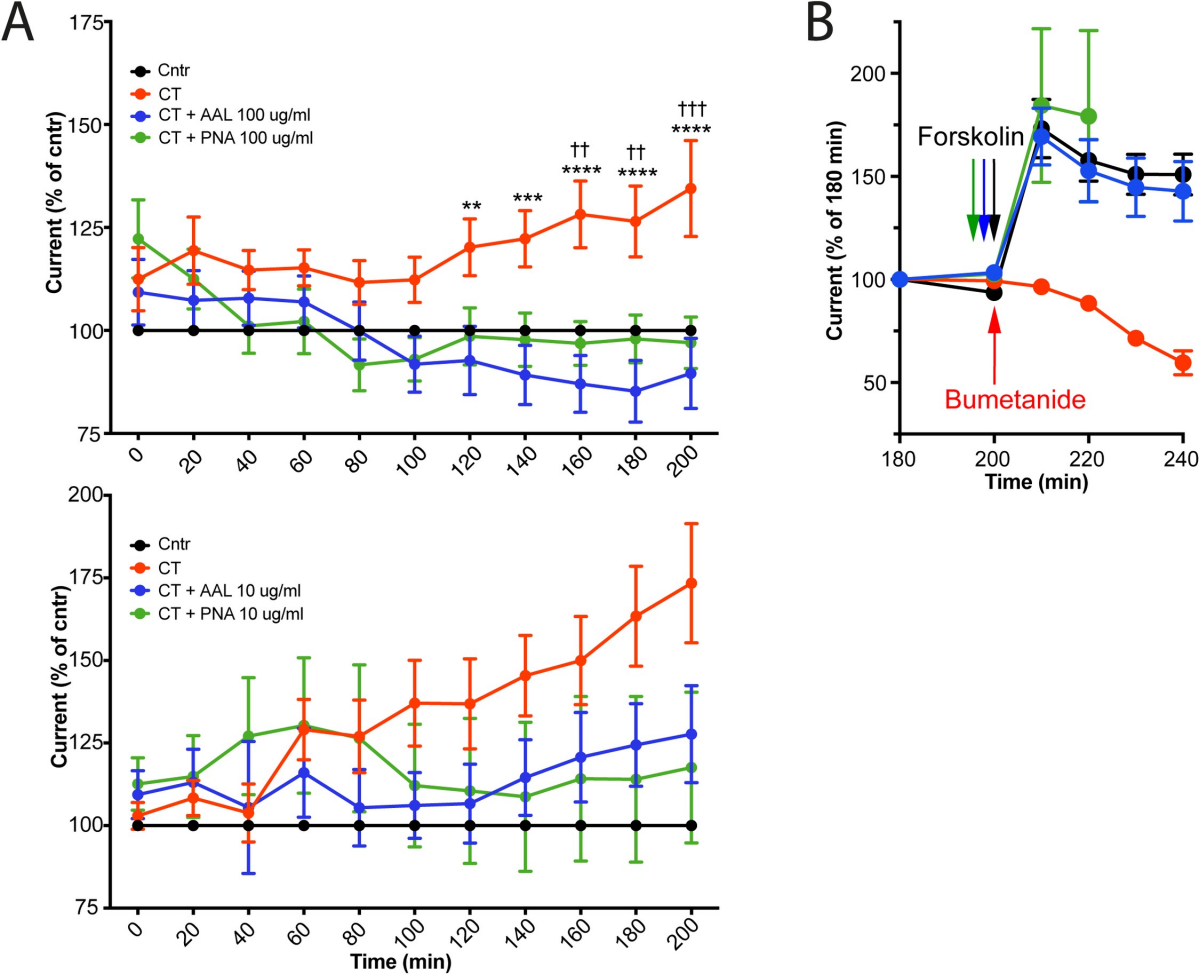
CT induced ion secretion can be inhibited by pretreating the tissue with AAL and PNA. Human jejunal mucosae were pre-incubated with or without AAL or PNA at the indicated concentrations, mounted in an Ussing chamber and exposed to CT. **(A)** Dot plot showing percent difference in I_ep_ to control tissue for jejunal mucosae over time. Each dot represents a mean of 4–7 donors (each treatment for each donor was tested in duplicates) with SEM error bars. Significance was calculated using a two-way-ANOVA with Tukey correction (compared to the CT). * represent CT to CT+AAL comparison and † represent CT to CT+PNA comparison (**** = p<0.0001 and ** = p<0.01). **(B)** Dot plot showing percent of start I_ep_ for jejunal mucosae at 180 min. AAL, PNA-treated or untreated tissue were treated with forskolin (or forskolin analog NKH477) bilaterally at 200 min. CT treated tissue were treated with bumetanide at 200 min. Each dot represents a mean of 2–3 donors (each treatment for each donor was tested in duplicates). Error bars show SEM.

To assess functional consequences of the binding of CT to different glycoconjugates, Ussing chamber experiments with human jejunal biopsies were performed. After removal of the muscular layer, the jejunal tissue was mounted in the chamber and CT added to the lumen-side of the jejunal tissue. After 140 minutes this resulted in an increased electrical current in the CT-treated tissue compared to PBS-treated control (Fig 6A). The active secretion of Cl^-^ by the CT-stimulated tissue was in control experiments confirmed by adding bumetanide after 200 minutes, a compound that inhibits stimulated Cl^-^ secretion through blocking Na+/K+/2Cl- transporter NKCC1 (Fig 6B). Pretreating the tissues on ice with AAL or PNA before mounting the tissues in the chamber completely inhibited the secretory response induced by CT (Fig 6A). Also a ten-fold lower concentration resulted in efficient inhibition of CT-induced secretion by both PNA and AAL (Fig 6A). Addition of forskolin (or forskolin analog NKH477) to AAL- or PNA-treated tissue resulted in a rapid and comparable secretory response to that observed in the untreated control tissue (Fig 6B). Although this control does not formally exclude the possibility that the binding of lectins to the cell membrane incapacitates the tissue from subsequent CT-mediated intoxication without directly blocking the binding of CTB to surface receptor, these results suggest that lectins binding to fucose- and galactose-carrying structures on human jejunal biopsies can interfere with CT-induced but not forskolin-induced secretion ex vivo.

### Inhibition of GSL synthesis or sialylation sensitizes glioma cells for CT-mediated intoxication

**Fig 7:**
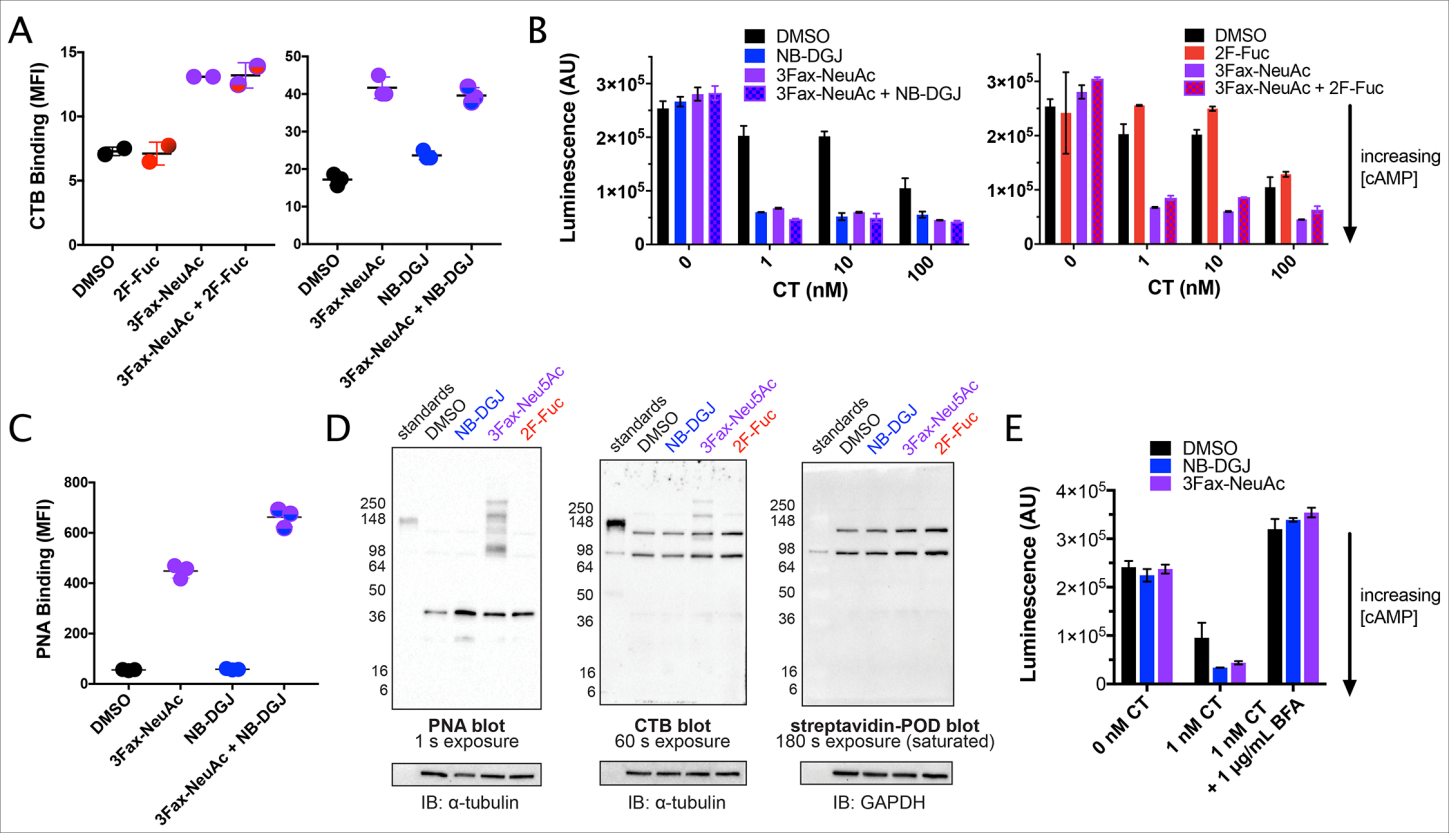
GM1-deficient C6 cells are sensitized to CT by inhibition of sialylation or GSL biosynthesis. **(A-E)** C6 cells were cultured with the indicated inhibitors for 72 h followed by: **(A)** Staining was then performed with biotin-CTB, followed by DTAF-streptavidin. Fluorescence was measured by flow cytometry, represented here by MFI. **(B)** 1 h exposure to CT after which accumulated cAMP was measured by the cAMP-Glo™ luminescence assay. Luminescence signal is inversely proportional to cAMP levels. **(C)** As in panel **A**, but stained with biotin-PNA, followed by DTAF-streptavidin **(D)** Cell lysates were separated by PAGE and probed with biotin-PNA, biotin-CTB, or no biotinylated reagent, followed by streptavidin-peroxidase conjugate and development with chemiluminescent substrate. Equivalent amounts of protein were loaded in each lane and blots were probed with an anti-α-tubulin or anti-GAPDH antibody to confirm equivalent loading. **(E)** As in panel **B**, but brefeldin A (BFA) was added 1 h prior to CT addition and was also present during CT induction.

Our results thus far show that interference with CT-mediated secretion can be achieved by pretreating human primary intestinal epithelial cells with lectins that bind fucosylated structures but not GM1 (AAL) or that bind terminal galactoses but with too low affinity to block GM1-binding (PNA) (Fig 6A, S1 Fig and S3 Fig) to CTB. We have previously shown that a human colonic epithelial cell line in which GM1 cannot be detected is still intoxicated by CT [20]. These results suggest that either non-GM1 GSLs or glycoproteins could act as functional receptors for CT. Rat C6 glioma cells do not express detectable levels of GM1, rather GM3 constitutes more 95% of the GSLs in these cells [38]. Consistent with prior reports [39], analyses of these cells also showed only a low level of binding to CTB and low sensitivity to CT-mediated cAMP induction (Fig 7A and 7B). To assess if this residual binding was due to GM3, the C6 cells were grown in NB-DGJ to block GSL synthesis. Unexpectedly, this treatment led to an increased sensitivity to CT-mediated intoxication (Fig 7B) and a slight increase in CTB binding (Fig 7A). Growing C6 cells in 3Fax-NeuAc, thereby blocking sialyltransferase activity, which is also required for GM3 biosynthesis, led to increased binding of CTB and increased cAMP levels following CT treatment (Fig 7A and 7B). Combination of the two treatments (NB-DGJ and 3Fax-NeuAc) did not increase the level of sensitivity compared to either treatment alone (Fig 7B). The increased sensitivity of C6 cells to CT following culture with the inhibitors was not due to effects on adenylate cyclase or the CFTR channel as forskolin induced an equivalent response in 3Fax-NeuAc and NB-DGJ treated cells as DMSO controls (S6 Fig). The level of staining obtained by fucose-binding lectin AAL in a lectin blot of C6 cell lysates was very low compared to human colonic epithelial cell line T84 (S6 Fig). Accordingly, growing the C6 cells in 2F-Fuc to reduce fucosylation resulted in a minor reduction in sensitivity to CT either as single treatment or in combination with 3Fax-NeuAc (Fig 7B). 3Fax-NeuAc but not NB-DGJ treatment led to increased availability of terminal galactoses as detected by increased binding of PNA by flow cytometry (Fig 7C). A PNA blot of cell lysates revealed that at least some of these terminal galactose residues were displayed on proteins, and the same molecular weight range on a CTB blot showed increased levels of CTB-binding glycoproteins in lysates from C6 cells treated with 3Fax-NeuAc but not NB-DGJ (Fig 7D). AAL binding to C6 cells was reduced by 2F-Fuc treatment, but 3Fax-NeuAc and NB-DGJ had no significant affect on AAL binding. This result suggested that the ability of 3Fax-NeuAc and NB-DGJ to sensitize C6 cells to CT is not due to increased expression of fucosylated ligands for CTB (S6C Fig). Brefeldin A (BFA) treatment of C6 cells fully blocked the CT-mediated intoxication, in cells grown in 3Fax-NeuAc, NB-DGJ or media, revealing that also non-GM1 receptors traffic through the secretory pathway (Fig 7E). While sialic acid-containing GSLs such as GM3 were previously shown to bind CT, the presence of these GSLs does not promote intoxication, rather they instead reduce the CT sensitivity of C6 cells, possibly by competing glycoproteins for binding to CT. In contrast to primary human intestinal cells, the CTB-binding glycoproteins in C6 cells appear to be primarily terminally galactosylated, consistent with the low fucosylation in this cell line. Hence, these experiments show that non-GM1 receptors are present in C6 cells where GSL synthesis has been blocked or the level of terminally galactosylated glycoproteins is increased and that these receptors traffic through the secretory pathway.

### CT induces an intact diarrheal response in mice lacking GM1 and GM1 related GSLs

**Fig 8:**
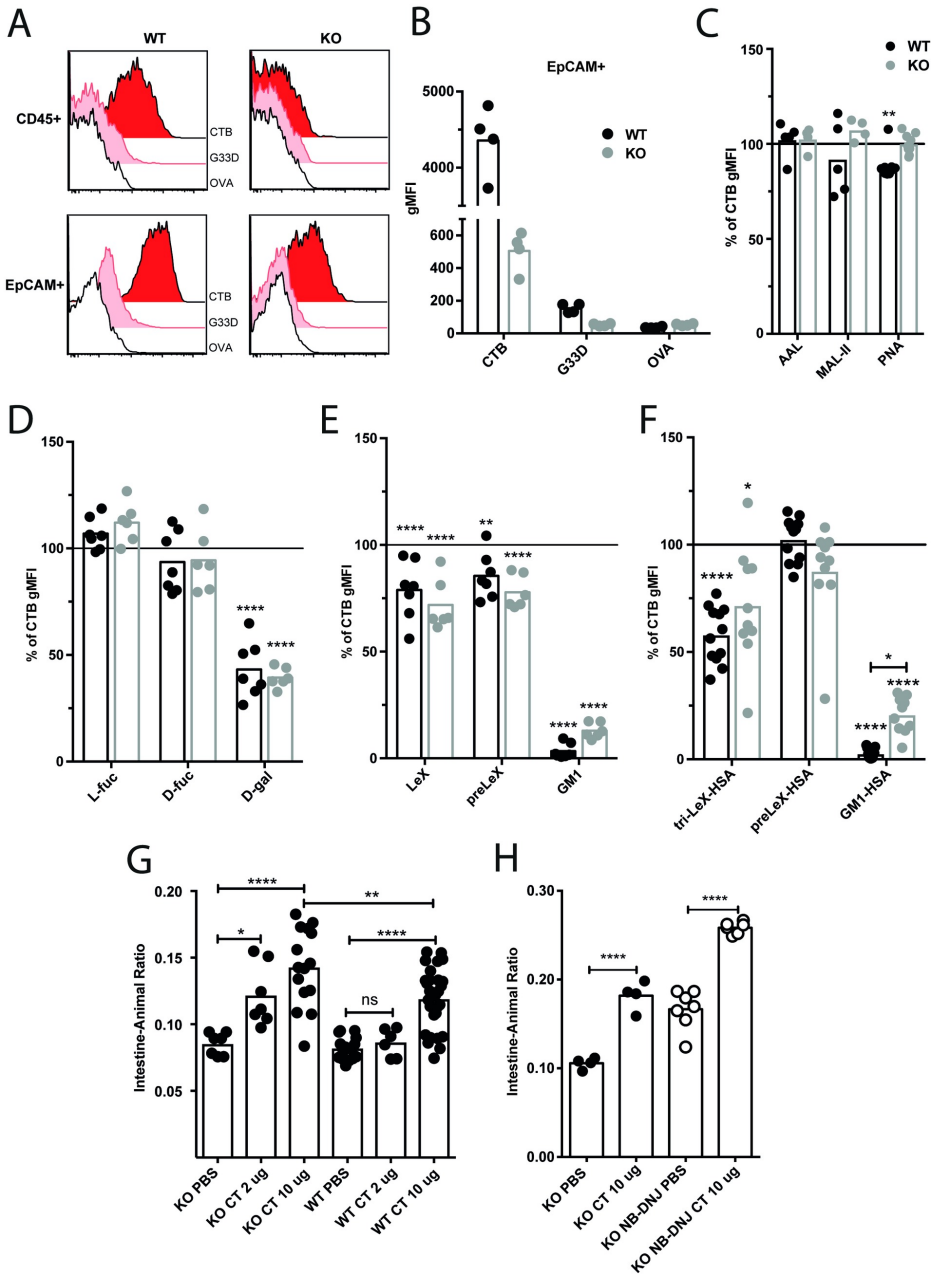
CT induces an intact diarrheal response in mice lacking GM1 and GM1 related GSLs. **(A)** Representative histograms from flow cytometry analyses of CTB-, G33D- and OVA-binding to WT and KO jejunal cells (non-villi epithelial and CD45+ cells). **(B)** Bar graph showing CTB, G33D and OVA gMFI of non-villi jejunal epithelial cells from wt (black) and KO (gray). **(C-F)** Bar graphs showing percent of gMFI of non-blocked CTB binding to non-villi jejunal epithelial cells (wt black and KO gray) following pretreatment of the cells with **(C)** lectins or CTB with **(D)** sugars, **(E)** oligosaccharides and **(F)** HSA-linked oligosaccharides. Graphs show the percent of gMFI of CTB binding to the cells where 100% represents CTB staining with no blocking. **(G-H)** Bar graph showing intestine-animal ratio (by weight) for WT and KO mice gavaged with PBS with or without CT. **(H)** KO mice were fed chow with (open circles) or without (full circles) NB-DNJ for 4 weeks prior to gavage. The graphs are from pooled experiments where each dot represents one animal (n = 4–30). Significance was calculated using a one-way-ANOVA with Tukey correction (**** = p<0,0001, *** = p<0,005, ** = p<0,01 and * = p<0,05).

To determine if binding to GM1 and GM1-related GSLs are required for CT to induce a diarrheal response, we took advantage of the B4galnt1 (KO) deficient mice [40], and made cell suspensions of jejunal tissues from KO and wt littermates. As expected, intestinal lamina propria leukocytes (CD45+) from wt but not from KO mice efficiently bound CTB (Fig 8A). In contrast, epithelial cells (EpCAM+) from KO mice stained with CTB but not as strongly as in wt mice (Fig 8A). The staining intensity of CTB was roughly ten-fold lower in KO than that of the wt mice, while the G33D mutant did not stain KO epithelial cells above background (Fig 8A and 8B). To determine if GM1 was still present in intestinal epithelial cells, lysates were analyzed by HPLC. GM1 was below the level of detection (10 fmol/mg tissue) in KO lysates and interestingly could not be detected in wt lysates either. Instead asialo-GM1 (GA1) was the most abundantly expressed GSL in wt intestine but this GSL was drastically reduced in KO mice (S7 Fig). Hence, with the exception of GM3, all previously described GM1-related GSLs reported to bind CTB were drastically reduced or below the level of detection in intestinal lysates from KO mice (S7 Fig). Immunoblotting with CTB on lysates revealed bands of high molecular weight (100-110kDa) as well as smaller (20kDa) (S7 Fig). Sub-fractionation of the lysates revealed the most prominent expression of CTB-binding proteins in the membrane fraction of both wt and KO mice (S7 Fig). Binding of CTB to glycolipids migrating in the dye front was only observed in the wt lysate, which further corroborates the HPLC analysis showing a drastic reduction in CTB-binding GSLs in KO intestinal lysates (S7 Fig). The binding of CTB to the blotted proteins was dependent on glycosylated structures, as treatment with periodate abolished this interaction (S7 Fig). These results show that GM1 cannot be detected in murine intestines and apart from GM3, CTB-binding GSLs are dramatically reduced in KO mice. In addition, glycoproteins that bind CTB are present in both wt and KO small intestines, while GSLs that bind CTB were detected only in wt small intestine.

To address the nature of CTB binding, blocking experiments with jejunal single cell suspensions were performed. In contrast to the results observed in human granulocytes and jejunal epithelial cells, neither AAL nor L-fucose blocked the binding of CTB-biotin to jejunal epithelial cells from wt or KO mice (Fig 8C and 8D). Le^X^-os, but also pre-Le^X^-os to a similar degree, only partially interfered with the binding of CTB to intestinal epithelial cells from the two strains of mice (Fig 8E). Coupling tri-Le^X^ (but not pre-Le^X^) to HSA resulted in a significant block of CTB-biotin binding to epithelial cells from wt and KO mice (Fig 8F). D-galactose, GM1-os and GM1-HSA each blocked the binding of CTB-biotin to epithelial cells from both wt and KO mice (Fig 8D and 8F). PNA blocked some of the binding of CTB to epithelial cells from wt but not KO mice while MAL-II had no effect (Fig 8C). No expression of Le^X^ by murine jejunal epithelial cells could be detected by flow cytometry although binding of AAL was readily observed (S7 Fig). These data show that the fucosylated structures expressed in KO mice are not crucial for the binding of CTB to epithelial cells, but galactose and GM1 can efficiently block this binding.

To finally assess if CT can induce a diarrheal response *in vivo* in the absence of GM1 and GM1-related GSLs, B4galnt1 KO and wt mice were gavaged with CT. Four to five hours later the severity of the intestinal secretory response was determined, similar to ligated loop method, by weighing the intestinal tissue including fluids and dividing it with the total weight of the animal [41,42]. This model of acute diarrhea revealed that KO mice as well as wt mice responded with a secretory response to CT (Fig 8G). In accordance with the results from NB-DGJ treatment of C6 cells, the severity of the response was significantly increased in the KO mice compared to wt mice (Fig 8G). In addition, the KO mice responded at lower doses than the wt mice (Fig 8G). These findings also make it highly unlikely that the response to CT in the KO mice is attributable to residual intestinal GM1 or GM1-related GSL expression caused by non-canonical biosynthesis of these GSLs. However, to further reduce remaining GSLs detected by HPLC, KO mice were given chow supplemented with n-butyldeoxynojirimycin (NB-DNJ, miglustat) that inhibits glucosylceramide synthase mediated addition of glucose to ceramide leading to reduction of all glycosylceramide-derived (including GM1-related) GSLs [43]. This treatment accordingly led to a further reduction of remaining GSLs including a 90% reduction in GM3 (S7 Fig). The effect of the NB-DNJ was also indicated by a known “side effect” of this treatment detected as increased fluid secretion into the lumen of NB-DNJ treated animals not receiving CT (Fig 8H). This is due to inhibition of sucrose isomaltase leading to increased fluid in the colon due to osmotic pressure. However, taking this shift in Intestine-Animal ratio into account, a gavage with CT resulted in comparable levels of secretory response in KO treated with NB-DNJ or not (Fig 8H). This shows that CT-mediated intoxication occurs in mice in which GM1 in intestinal tissues are below level of detection and all GM1-related GSLs previously shown to bind CTB are absent or reduced by 90%.

Collectively our data identify Le^X^ (CD15) as one, but not the only CTB-binding fucosylated moiety present on primary human granulocytes and jejunal epithelial cells. In addition, we show that inhibition of GSL synthesis or sialyltransferase activity render GM1-deficient cells, through a mechanism dependent on traffic through the secretory pathway, susceptible to CT-mediated intoxication. Finally, and in support of non-GM1 CTB-binding receptors *in vivo*, we show that B4galnt1 KO mice, lacking GM1 and GM1 related GSLs, present an increased diarrheal response to CT.

## Discussion

The events that occur after uptake of CT and lead to fluid secretion by intoxicated epithelial cells are well-characterized. Receptor-mediated uptake through GM1 has long been considered to be the event that initiates this intracellular cascade. However, we recently showed that CT also binds fucosylated structures on human colonic epithelial cells [20]. In this study, we extend this finding and identify that CT binds Le^X^ when this moiety is linked to proteins (tri-Le^X^) but not to ceramide (Le^X^) and that this binding is dependent on the fucose in Le^X^. Furthermore, inhibition of GSL synthesis in GM1-deficient cells increases sensitivity to CT, indicating a role for glycoproteins. Finally, we show that mice lacking GM1 and GM1-related GSLs present an increased diarrheal response to CT, demonstrating that functional receptors other than GM1 exist and function *in vivo*.

The data presented here confirm the importance of fucosylated structures in CTB binding to human intestinal epithelia, while also indicating that non-fucosylated structures may contribute to CTB binding, particularly in rat and mouse cells. Using immunoprecipitations of human colonic cell lines and ELISAs, we show that CTB binds to glycoproteins that are modified with the fucosylated glycan Le^X^, and that binding of CTB to human intestinal epithelial cells can be blocked by fucosylated structures. We note that some human primary jejunal epithelial cells do not express Le^X^, but can still bind CTB indicative of either GM1-mediated binding or additional glycosylated receptors. Using wt and B4galnt1 KO mice, we observe that binding of CTB to white blood cells is mediated via either GM1 or GM1-related GSLs but on intestinal epithelial cells additional receptors exist. Binding of CTB to mouse intestinal epithelial cells can be blocked by galactose or with GM1-os, and the G33D CTB mutant does not bind to the receptors on these cells. These results suggest that these non-GM1 receptors bind CTB in the same site as GM1. However, and in contrast to human intestinal epithelial cells, CTB binding to these murine receptors cannot be blocked by interference with fucosylated structures.

Differences in fucosylation could underlie the species-specific differences in CTB binding that we observe. A general lower total level of fucosylated structures in the murine compared to human intestine seems unlikely as the fucose-binding lectin AAL efficiently labels primary murine jejunal cells. Hence, it rather seems that the fucose linkage (α1–3, α1–2 or α1–6) and the nature of the aglycone (protein or ceramide) could explain the difference between the binding of CTB to murine and human cells [44]. Fucosylation in intestinal epithelial cells is mediated by several fucosyltransferases (FUTs). The expression of the different FUTs that jointly determine fucosylation is different in mouse and man and also differs among human individuals. Some FUT expression has also recently been shown to be driven by bacterial colonization [45–47]. It is known that transcripts of FUT4 and 9, the enzymes that add α1-3-linked fucose and are required to synthesize Le^X^, are in adult mice only expressed at low levels in the small intestine [48,49] and accordingly we cannot detect Le^X^ on murine jejunal epithelial cells by flow cytometry.

Determining which site of CT engages in binding to epithelial cells could be of importance not only for potential blocking therapies but also for generation of detoxified versions of CT to be used as adjuvants. Crystallography and mutational analysis of CTB has identified that the Gly33 residue plays a crucial structural role in creating a binding pocket for the sialic acid of GM1 [35]. Interestingly, we observed that the G33D mutant also showed a drastic reduction in binding to protein-linked Le^X^, human granulocytes and primary jejunal epithelial cells, implying that Le^X^ binds to the same (or similar) site as GM1. However, combining saturated levels of GM1-os and unsaturated levels of Le^X^-os blocked CTB binding to granulocytes significantly more than saturated levels of GM1-os alone. Taken together, these data suggest the possibility that CTB may have more than one binding sites for Le^X^ and Le^X^-like structures. Indeed, crystallography studies of the closely resembling HBGA Le^y^ identified binding to CTB in a site distant from the GM1 binding pocket [21]. The study did not report binding of HBGAs close to the “GM1-site” (involving Gly33) as suggested by our results, which could possibly be explained by structural differences between Le^X^ and Le^y^. Alternatively, the galactose binding in the “GM1-site” (remaining from purification of CTB in this study) could have blocked this site during the co-crystallization.

Several polymers and polymer-like molecules have been tested for inhibition of CTB (and LTB) binding but most of them were tested in a GM1-based ELISA [50–57]. We here present data suggesting at least one additional binding site on CTB, that cannot be blocked by GM1, showing that the aforementioned molecules would benefit from being screened for inhibiting binding of CTB to human intestinal epithelial cells (with fucosylated structures) before assessment of their efficacy to block holotoxin-mediated cAMP induction and ion secretion. Intestinal organoids have recently been used for this purpose [58]. However, using this system, CT binds to the basolateral side, which might not fully mimic the *in vivo* setting.

Early studies in larger animals and human volunteers have indicated that the majority of CT-induced fluid secretion occurs in the small intestine while the colon contributes very little to the diarrhea [59–61]. The human small intestine expresses very low levels of GM1 as well as asialo-GM1 and fucosyl-GM1 has not been detected [8,15]. We here show that interference with fucosylated structures using the lectin AAL efficiently blocks CTB binding to human jejunal cells. Although multiple (non-GM1) fucosylated receptors may exist and their levels vary between individuals, we show by antibody-mediated blocking that Le^X^ is a receptor for CTB. This is in line with our previous study that showed that in the T84 cell line CEACAM5 binds to CTB. Members of the CEACAM (CD66) family can be modified with Le^X^ and CEACAM1 on neutrophils has been shown to bind lectins via Le^X^ [62]. In preliminary experiments, we have observed that CTB also binds sialyl-Le^X^ and Le^y^ that (like Le^X^) contain α1–3 linked fucose. Binding to Le^X^-negative epithelial cells is also dramatically reduced with the G33D CTB mutant. Blocking reagents that bind to the vicinity of the aa 33 can therefore inhibit binding to GM1 as well as Lewis antigens. Hence, selectively interfering with either a GM1 or Le^X^ driven pathway in human primary cells by pretreating CT with glycoconjugates is at present not possible. However, the potent abilities of AAL (that does not bind GM1 or GM1-related GSLs) and PNA (that doesn’t bind GM1 with enough affinity to block binding to CTB) to block CT-mediated ion secretion by human jejunal cells indicates that non-GM1 receptors are of importance for CT-mediated intoxication. Our results with B4galnt1 KO mice and C6 cells, where blocking of GSL synthesis renders the cells more sensitive to CT-mediated intoxication, would argue that these non-GM1 receptors are glycoproteins rather than GSLs. Whether these glycoprotein receptors are fucosylated and/or have terminal galactoses will be dependent on cell types and species where for example human intestinal epithelial cells are heavily fucosylated while rat glioma cells are not.

In summary, we have in this study added to our previous work identifying functional consequences of CT binding to fucosylated structures on human colonic cell lines [20] by showing that *in vivo*, in the absence of GM1 and all previously described ceramide-linked binders of CT, diarrhea following ingestion of CT still occurs. In addition, our data indicate that in a GM1-deficient context, an additional reduction in GSLs can lead to increased sensitivity to CT-mediated intoxication in vitro and in vivo. We identify the α1,3 linked fucose of Le^X^ as a novel binder of CTB expressed by human primary jejunal epithelial cells and granulocytes. The aglycones to which Le^X^ and other non-GM1 related receptors for CTB are attached, as there are most likely several (proteins and/or GSLs), and the mode of subsequent cell intoxication (autonomous or collaborative) remain to be determined. Overall, the discovery of Le^X^ and other non-GM1 related receptors for CT opens up avenues for new blocking therapies and design of detoxified enterotoxin-based adjuvants.

## Materials and methods

### CTB and blocking reagents

Recombinant CTB and G33D-mutant were produced in-house as described before [63]. CTB, G33D and OVA were conjugated to biotin or HRP as described below.

For blocking CTB binding, different compounds were used. L-fucose, D-fucose and D-galactose (used at 50 mM if nothing else stated, cat nos: F2252, F8150 and G0750, Sigma-Aldrich) or Le^X^-os, pre-Le^X^-os (N-Acetyl-D-lactosamine/LacNAc) and GM1-os (GLY049, GLY008 and GLY096, Elicityl) or HSA linked mono- or tri-Le^X^, pre-Le^X^ and GM1 (product no 61/04, 61/56, 61/70 and 61/69, IsoSep) were used to pre-block CTB or G33D. CTB was also blocked by pre-incubating cells with the lectins AAL (*Aleuria Aurantia* lectin binding fucosylated structures), PNA (Peanut agglutinin) and MAL-II (*Maackia Amurensis* lectin II binding structures with sialic acid) (product no L-1390, 1070 and L-1260, Vector Laboratories).

### Isolation of cells from human blood

Blood donated anonymously through Sahlgrenska University hospital blood bank was used to investigate binding of CTB, G33D and OVA-biotin (biotinylated using the kit Lightning-Link Rapid Biotin, Innova) to different blood cells from donors of different A,B, AB or O-blood group. Since obtained results could not be traced back to a specific individual, ethical approval was not needed according to Swedish legislation (4§, SFS 2003:460).

The majority of RBCs were lysed using ACK-buffer (Gibco, Thermo Scientific) according to the manufacturer’s instructions. The cells were stained with mAbs against CD45-APC-H7, CD3-FITC, CD19-BV510, CD66a/c/e-PE (Biolegend), CD14-PE-Cy7, CD56-BV711 (BD Biosciences) and CTB, G33D or OVA-biotin followed by streptavidin-BV421 and the live/dead marker Zombie Red. Cells were then analyzed using flow cytometry on an Aria-III (BD Biosciences) and the data analyzed using FlowJo software (Version 9.7.6, Three Star).

Pure granulocytes were obtained by Ficoll-Hypaque (GE Healthcare, Sigma-Aldrich) density gradient centrifugation of human whole blood as described elsewere [64]. In short, buffy coats were obtained from healthy blood donors and mixed 1:1 with 2% Dextran T500. After sedimentation of RBCs, the upper phase was separated into granulocytes and mononuclear cells by density gradient centrifugation. Residual erythrocytes in the pellet were lysed in distilled water to yield a pure population of granulocytes.

RBCs for ELISA were purified by diluting human blood 1:10 in PBS and spinning at 300*g* for 5 min. The supernatant together with the leukocytes on top of the RBCs were removed and the RBC pellets were frozen.

Cells not used in flow cytometry were spun down and pellets were frozen and then lysed using RIPA-buffer (50 mM TrisHCl, pH 8.0, 150 mM NaCl, 1% NP-40, 0.5% sodium deoxycholate, 0.1% SDS and a protease inhibitor (Roche, Sigma-Aldrich) to be used in sandwich ELISA.

### Isolation of human enterocytes

Patients undergoing gastric bypass surgery donated human jejunum resections after informed consent. The resected tissue was immediately put in ice-cold Krebs-Ringer solution (NaCl 118 mM, KCl 4.69 mM, CaCl 2.52, MgSO_4_ 1.16 mM, NaH_2_PO_4_ 1.01 mM, NaHCO_3_ 25 mM and D-glucose 12.2 mM). Before transport, small pieces of mucosa were dissected from the tissue and incubated in Krebs-Ringer solution for 45 min on ice with or without 100 μg/ml AAL. The pieces were then mounted in an Ussing chamber as described below. After transport, the rest of the mucosa was dissected from the muscle layer, chopped into small pieces and incubated 3 x 15 min at 37°C in HBSS (without Ca^2+^ and Mg^2+^, Gibco, Thermo Scientific) with 5 mM EDTA, 2% FBS (Gibco, Thermo Scientific) and 15 mM HEPES (*Fisher* BioReagents). The mucosa was then washed with HBSS without EDTA for 10 min at 37°C followed by enzymatic degradation in R10 medium (RPMI1640, *Lonza*, with 10 mM HEPES, 10% FBS, 2 mM L-glutamine 1 mM Na-pyruvate, 1% PenStrep, *Fisher BioReagents* and 10 μg/ml gentamicin, Gibco, Fisher Scientific) with Liberase (with medium level termolysin) and DNAse I (Roche, Sigma-Aldrich) together with 5 mM CaCl_2_ for 45 min at 35°C. The cell suspension was filtered, washed and stained with mAbs against EpCAM-FITC, CD45-APC-H7, CD66a/c/e-PE (Biolegend), Le^X^-BV786 (BD Biosciences) and with CTB/G33D/OVA-biotin (conjugated with kit from Innova) and streptavidin-BV421 and the live/dead marker Zombie Red (Biolegend). Blocking of CTB binding was done as above. The cells were then analyzed using flow cytometry. Leftover cells were spun down at 350*g* and frozen. They were then lysed using RIPA-buffer to be used in sandwich ELISA. Epithelial cells from colonic tissue (healthy tissue from patients undergoing surgical tumor removal) were isolated as previously described [20].

The Ethical Review Board, Gothenburg, Sweden, gave ethical approval for acquisition of and experiments on these tissues (jejunal tissue: no 261–13 and colon tissue: no 249–15).

### Mice

Mice -/- or +/- for the GalNAcT-gene (C57BL/6 background) were kindly donated by Professor Ronald L. Schnaar (Johns Hopkins University School of Medicine, MD, USA). The -/- mice are deficient in complex (and some non-complex) GSLs, as confirmed previously [40,65]. The mice were bred (heterozygous breeding) and maintained in individually ventilated cages in the animal facility at Sahlgrenska Academy, University of Gothenburg, Sweden. +/- mice express similar levels of GM1 as +/+ mice and were therefore used as (wt) controls [66]. All animal experiments were performed in accordance with approved ethical permits granted by the regional Animal ethics committee (Ethics no: 150/15).

### Isolation of cells from murine tissues

The jejunal section from mouse intestine was excised along with the spleen, and peripheral blood was collected. The enterocytes were isolated using the same protocol as seen above but without initial removal of the muscular tissue. The cells were stained with mAbs against CD45.2-PerCP-Cy5.5, EpCAM-FITC, CD3-APC, CD66-PE and CTB, G33D and OVA-biotin to evaluate CTB binding to epithelial and subsets of hematopoietic cells. Anti-LeX-BV786 was also used in some samples to detect Le^X^ on the enterocytes.

Spleen cells were isolated by gently mashing the spleen through a 100-micron mesh filter and the majority of RBCs were then lysed using RBC lysing buffer (BD). Murine blood was also collected in heparinized tubes (Sarstedt) and RBCs were lysed in the same way. The cells were stained with mAbs against CD45.2-PerCP-Cy5.5, CD3-APC, CD19-FITC, Ly6G-Af700 and CD115-PE as well as CTB, G33D and OVA-biotin followed by streptavidin-BV421 and the live/dead marker Zombie Red to characterize CTB binding to different cell types. Cells from the intestine were also lysed as above and used for western blot for CTB as described below. Sub-fraction lysing was done using Subcellular Protein Fractionation Kit (Cat no: 78840, Thermo Fisher) according to manufacturers instructions. If membrane sections were to be treated with periodate, membranes were after blocking washed for 5 minutes with PBS before adding the periodate solution (50 mM NaIO4 in PBS). Membrane sections were then incubated for 20 minutes in room temperature in darkness. After incubation, periodate solution was discarded and membrane sections were washed in PBS, and then washed with a 0.1% Tween 20 in PBS solution before staining with CTB.

### In vivo effect of CT in mice

Transgenic mice +/- or -/- for the GalNAcT-gene as described above were fed with 10 μg CT (Azide free, List Laboratories) in PBS with 3% NaHCO_3_. The mice were sacrificed after 4–5 h and the weight of the whole intestine (including accumulated fluid) was compared to the full body weight of each mouse [42]. Control mice were fed only PBS with 3% NaHCO_3_. KO mice were also fed standard chow supplemented with NB-DNJ (600 mg/kg/day) for 3–4 weeks to eliminate remaining GSLs and then subjected to the same treatment with CT as above. Tissue from the small intestine of wt and KO mice were also harvested for HPLC-analysis of GSL content.

### Ganglioside isolation, purification and quantification

Murine small intestinal tissue was homogenized in deionized water (100 mg wet weight/ml) with a glass/Teflon homogenizer. The tissue was then subjected to GSL extraction, purification and fluorescent labeling as described previously [67]. Briefly, GSLs were extracted with chloroform/methanol overnight. Total GSLs were recovered using SepPak C18 columns, eluted with CHCl_3_/MeOH and the samples were dried under nitrogen. After ceramide glycanase digestion of the dried lipid extracts to release the glycan moiety, samples were labeled with anthranilic acid. Derivatized oligosaccharides were purified on Spe*-*ed Amide*-2* columns (Applied Separations, Allentown, PA). Normal phase-HPLC (NP-HPLC) was carried out on a Waters 2695 separations module with a Waters 2475 fluorescence detector (Waters Corporation, Hertfordshire, UK). A TSK-amide 80 NP-HPLC column (4.6 × 250 mm, Tosoh Biosciences, Sigma-Aldrich, UK) was used for the separation and maintained at 30°C using a Waters column heater. The solvents and gradient conditions were as previously described [67]. The peak area per femtomole of GSL-derived oligosaccharide present was determined and used to quantify the concentration of GSLs in the tissue samples.

### Photocrosslinking and western blot

For crosslinking experiments, 1.8 million HL-60 cells were seeded into 10 cm tissue culture dishes with media containing 100 μM Ac_4_ManNDAz. When used, glycosylation inhibitors were also added at the time of seeding to achieve these final concentrations: 50 μM NB-DGJ, (N-(n-Butyl)deoxygalactonojirimycin; Santa Cruz Biotechnology, Cat No. sc-221974), 1 μg/mL kifunensine (Sigma-Aldrich, Cat No. K1140), 2 mM benzyl-α-GalNAc (Sigma-Aldrich, Cat. No. B4894), and 200 μM 2F-Fuc (2-Fluoro-peracetyl-fucose; EMD Millipore, Cat. No. 344827). After culturing for 72 h, cells were harvested by centrifugation and resuspended in fresh media at a density of 2.5 million cells/mL with 1.25 μg/mL CTB (Sigma-Aldrich, Cat. No. C9903). Cells were incubated for 45 min at 4°C in the dark to allow binding to occur, then the “minus UV” plates were kept at 4°C for an additional 45 min, and the “plus UV” plates were irradiated on an ice/water bath for 45 min at 365 nm (UVP, XX-20BLB lamp). Cells were harvested by centrifugation and washed twice in PBS and then lysed in RIPA-buffer. Lysates were incubated on ice for 30 min, then centrifuged at 14,000*g* for 10 min at 4°C. The supernatant was collected, treated with 4X SDS loading dye for 5 min at 90°C, and loaded into a single well of a 6% or 15% Tris-glycine gel. After separation and transfer to PVDF membranes, the blots were probed with 1:5000 diluted anti-CTB (Abcam, Cat. No. ab34992) in 4% non-fat milk/TBS including 0.1% Tween-20 at 4°C overnight. After three washes with TBST (and subsequent staining with secondary anti-rabbit-IgG-HRP for CTB detection), the membranes were washed and incubated with SuperSignal West Femto Maximum Sensitivity Substrate for 1 min, then imaged with a ChemiDoc MP Imaging system (Bio-Rad).

### Immunoprecipitation and western blot

HL-60 cells were used as a model for human granulocytes and T84 as a model for human intestinal epithelial cells. Lysates from HL-60 or T84 cells were used in SDS-PAGE and Western blot with or without prior immune-precipitation (IP).

For IP with the anti-CTB antibody, the CTB-bound lysates were diluted to 1 mg/mL in RIPA buffer, and then 350 μg HL60 lysates or 500 μg T84 lysates were added to 1.5 mL microcentrifuge tubes. Then, 0.1 μg of either ant-CTB antibody or normal rabbit IgG (EMD Millipore, Cat. no. NI01) was added per 50 μg of lysate, and the samples were mixed by end-over-end rotation overnight at 4°C. The lysate/Ab mixture was then added to 10 μl of TrueBlot anti-rabbit Ig IP beads (Rockland Immunochemicals, Cat. no. 00-8800-25) and mixed by end-over-end rotation for 2.5 h at 4°C. The beads were washed four times with 200 μL RIPA buffer, and eluted with 10 μl 2X SDS loading dye for 5 min at 90°C. The supernatant was collected and loaded into a single well of a 6% Tris-glycine gel. After separation and transfer to PVDF membranes, the blots were probed with 1:2000 diluted anti-Le^X^ (HI98, BD Biosciences, Cat. No. 555400) in 3% BSA/TBS including 0.1% Tween-20 at 4°C overnight. After 3 washes with TBST (and secondary anti-mouse-IgM-HRP for Le^X^ detection), the membrane was incubated with SuperSignal West Pico Chemiluminescent Substrate for 2 min, then imaged with a ChemiDoc MP Imaging system (Bio-Rad).

### ELISA

ELISA was performed using different concentrations of HSA-linked oligosaccharides (mono- or tri-Le^X^, pre-Le^X^ and GM1, IsoSep) which were immobilized in 96-well micro plates at RT overnight (MICROLON 600 High Binding, Greiner, VWR) and subsequently blocked with 0.2% BSA (Sigma-Aldrich) in PBS at 37°C. The plates were incubated with CTB or G33D linked to HRP (conjugated using the Lightning-Link HRP-kit, Innova) with or without blocking sugars or pretreating wells with lectins or antibodies in PBS + 0.2% BSA and 0.1% Tween20 (Sigma-Aldrich) at RT. To show lectin binding, biotinylated lectins were used to probe immobilized HSA-os and then detected using streptavidin-HPS (RnD).

In the second mode, a sandwich-approach was used by immobilizing anti-Le^X^ mAb (HI98, Biolegend) with subsequent blocking with 0.2% BSA in PBS at 37°C. Lysates from human tissues (isolated cells lysed in RIPA buffer) were then incubated at RT with light shaking, washed with PBS + 0.1% Tween20 and incubated with CTB-HRP as above.

Detection for both modes were done using o-phenylenediamine dihydrochloride (OPD, Sigma-Aldrich) (1 mg/ml) dissolved in 0.1 M citrated buffer pH 4.5 for 15 min after which H_2_SO_4_ was added to stop the reaction. The plates were then read using Lx800 (BioTek) and absorbance values obtained. Background values (absorbance in wells coated only with BSA but probed with CTB-HRP) were subtracted.

### RIA

A radioimmuno assay (RIA) was also employed to detect binding of CTB to GM1 or Le^X^ covalently linked to ceramide, i.e. to glycosphingolipids, in microtiter wells [68]. In short, 50 μl of serial dilutions (each dilution in triplicate) of pure glycosphingolipids in methanol were applied to microtiter wells (Falcon 3911, Becton Dickinson Labware). When the solvent had evaporated, the wells were blocked for 2 h at room temperature with 200 μl of PBS with BSA (2% w/v). Thereafter, the wells were incubated for 2 h at room temperature with 50 μl of ^125^I-labeled CTB (labeled with a kit by the Iodogen method according to the manufacturer’s (Pierce, Rockford, IL) instructions) diluted to 2 x 103 cpm/μl in BSA/PBS. After washing 6 times with PBS, the wells were cut out and the radioactivity counted in a gamma counter.

### Ussing chamber

The dissected mucosae were mounted in mini-Ussing chambers that had a biopsy insert with a diameter of 2 mm and a square area of 0.034 cm^2^ (*Warner instruments*). Both the luminal and the serosal side were bathed in 5 mL Krebs solution (as described above) and were continuously oxygenated and stirred with 95% O_2_ and 5% CO_2_ gas flow at 37°C. At the serosal side, glucose is added in the Krebs solution to provide an energy substrate, while in the mucosal bath mannitol is added to maintain osmotic balance across the mucosa. In general six Ussing chambers were mounted per individual (2 PBS controls, 2 receiving 2 ug/ml of CT and 2 with tissue incubated in 100 ug/ml AAL for 1h on ice (also receiving 2 ug/ml of CT)).

Potential difference (PD) was measured with a pair of matched calomel electrodes *(*REF401, Radiometer analytical). Square wave analysis (the Ussing Puls Method, UPM) was used to determine the tissue’s epithelial electrical resistance (R_ep_). The epithelial net ion current (I_ep_) was obtained using Ohm’s law (I_ep_ = PD/R_ep_). The UPM has the advantage of estimating specifically R_ep_ and is described in detail elsewhere [69]. Briefly, the method is based on the concept that the epithelium acts as a capacitor and resistor coupled in parallel. Short current pulses charge the epithelial capacitor and when the current ends, the capacitor is gradually discharged. The epithelial voltage response as assessed from the discharge curve and the magnitude of the applied current were used for calculation of R_ep_. All electrical recordings were collected using specially constructed amplifiers connected to a computer with customized software developed in LabView (National Instruments) for online visualization, data storage and analysis. To be eligible for experimentation, each preparation had to fulfil certain viability criteria before baseline. The jejunal specimens thus had to have a lumen negative PD of ≥1,8 mV and R_ep_ of ≥6 Ω^.^cm^2^.

#### Experimental procedures

After a baseline period of 15 minutes, the CT (2 ug/ml) or PBS control was added to the luminal compartment and then run for 200 minutes. To ensure that ion transport was active (and that the tissue was still alive) forskolin (10 uM, *Sigma-Aldrich*) or forskolin analog NKH477 (10 uM, *Sigma-Aldrich*) or a chloride co-transporter inhibitor bumetanide (10–4 mol/L, *Roche*, *Sigma-Aldrich*) were added bilaterally at 200 min.

### Flow cytometry, lectin blots, and cAMP assay with C6 cell line

C6 cells were obtained from the ATCC and cultured in F-12K Medium (Kaighn's Modification of Ham's F-12 Medium) (ATCC, catalog no. 30–2004) containing 2.5% FBS (Thermo Fisher Scientific, catalog no. 16000044) and 15% Horse serum (ATCC, catalog no. 30–2040).

#### Flow cytometry

10 ml of C6 glioma cells were seeded in 10 cm tissue culture plate at a density of 0.4 M cells/ml. Inhibitors were added to achieve the following concentrations: 100 μM NB-DGJ, 200 μM 3Fax-NeuAc (3-Fluoro-peracetyl-NeuAc; EMD Millipore, Cat. No. 566224), or 200 μM 2F-Fuc. Experimental samples were compared to the appropriate vehicle-only control; DMSO for 3Fax-NeuAc or 2F-Fuc and water for NB-DGJ. After culturing for 72 h, cells were harvested by adding 4 ml of 1 mM EDTA in DPBS and incubating the cells for 20 min at 37°C after which the cells were dislodged by gentle resuspension. The cells were washed twice by resuspended in DPBS, centrifuged at 500*g* for 3 minutes and EDTA removed by aspiration. The cells were then resuspended in DPBS containing 0.1% (w/v) BSA (DPBS/BSA) and 0.75–0.8M cells were added per well to the V-bottom plate (Costar, Cat. no. 3897) and the cells were washed twice by resuspension in 200 μl cold DPBS/BSA followed by centrifugation at 730*g* for 5 min at 4°C. Cells were then incubated for 30 min on ice with diluted biotinylated CTB (Thermo Fisher Scientific/Invitrogen, Cat. No. C34779) or biotinylated lectins (Vector Labs: PNA, Cat. B-1075; MAL-II, Cat. No. B-1265; AAL, Cat. No. B-1395) at the following concentrations: 100 μl of 10 μg/ml was added per well. The cells were washed twice with 200 μl of cold DPBS/BSA and incubated for 30 min on ice with 100 μl of 7.7 μg/ml dilution of fluorescein (DTAF) streptavidin (Jackson ImmunoResearch; Cat. No. 016-010-084) added per well. The cells were again washed twice, stained with propidium iodide (PI) (Sigma-Aldrich; Cat. No. P4170) at a final concentration of 2.5 μg/ml and analyzed on a FACSCalibur flow cytometer (BD Biosciences, UT Southwestern Flow Cytometry Core facility). Dead cells were excluded based on PI staining on the FL3 channel and the fluorescence intensity of the live population was determined on the FL1 emission channel.

#### Lectin blots

For comparison of fucosylation among different cell lines, C6 cells or T84 cells were washed twice with Dulbecco’s Phosphate Buffered Saline (DPBS), and lysed in RIPA buffer (50 mM Tris-HCl, pH 8.0, 150 mM NaCl, 0.1% (w/v) SDS, 0.5% (w/v) sodium deoxycholate and 1% (w/v) IGEPAL CA-630) with protease inhibitor cocktail (Sigma-Aldrich/Roche, Cat. No. 11836170001). After vortexing, lysate was incubated on ice for 30 min and centrifuged at 20817*g* for 10 min. Supernatant was either directly used for further analysis or frozen in liquid nitrogen and stored at -80°C. Protein concentration was determined with a Pierce BCA Protein Assay Kit (Thermo Fisher Scientific, Cat. No. 23225) and BSA was used as standard.

For C6 cells treated with various inhibitors, 250,000 cells were plated in 6-cm tissue culture plates in 5 ml media and 5 μl of DMSO, NB-DGJ (100 mM stock), 3Fax-NeuAc (200 mM stock) or 2F-Fuc (200 mM stock) was added. After 72 h of culturing, cells were washed twice with DPBS, and lysed as above.

For lectin blots, 10 μg of lysate from each sample were diluted in deionized water and 4 x SDS loading dye (200 mM Tris-HCl, pH 6.8, 8% (w/v) SDS, 0.08% (w/v) Bromophenol blue, 40% (v/v) glycerol, and 40 mM DTT) was added. Lysate was denatured at 90°C for 5 min and centrifuged at 17000*g* for 1 min before being loaded on a 12% TGX Stain-Free gel (Bio-Rad, Cat. No. 1610185). Gels were activated and imaged with a ChemiDoc MP Imaging system (Bio-Rad). Proteins were transferred to PVDF membrane at 87 mA, 4°C overnight and blots were blocked with 1x Carbo-Free blocking solution (Vector Laboratories, Cat. No. SP-5040) in TBST (10 mM Tris-HCl, pH 8.0, 150 mM NaCl, 0.1% (v/v) Tween-20). For AAL, PNA and CTB blot, blots were incubated with biotin-AAL (2 mg/ml stock, Vector Laboratories, Cat. No. B-1395), biotin-PNA (5 mg/ml stock, Vector Laboratories, Cat. No. B-1075) or biotin-CTB (1 μg/μl stock, Thermo Fisher Scientific/Invitrogen, Cat. No. C34779) at 1:1000 dilution in 1x Carbo-Free blocking solution in TBST at 4°C overnight and washed with TBST before being incubated with streptavidin-POD (500 U/ml stock, Sigma-Aldrich/Roche, Cat. No. 11089153001) at 1:4000 dilution at room temperature for 1 h. For Streptavidin-POD blot, blot was directly incubated with streptavidin-POD (1:4000 dilution) at room temperature for 1 h after blocking. All blots were developed with SuperSignal West Pico Chemiluminescence Substrate (Thermo Fisher Scientific, Cat. No. 34080) with a ChemiDoc MP Imaging system.

Blots were incubated in mild stripping buffer (200 mM glycine, 0.1% (w/v) SDS, 1% (v/v) Tween-20, pH 2.2) at 37°C for 45 min and incubated with anti-histone H3 antibody (Abcam, Cat. No. ab1791, 1:2000 dilution, for AAL blot), anti-α-tubulin antibody (Sigma-Aldrich, Cat. No. T6199, 1:10000 dilution, for PNA and CTB blot), or anti-GAPDH antibody (Abcam, Cat. No. ab8245, 1:10000 dilution, for streptavidin-POD blot) at room temperature for 1 h and washed with TBST. The blots were then incubated with HRP conjugated goat anti-rabbit IgG (H+L) secondary antibody (Thermo Fisher Scientific, Cat. No. 65–6120, 1:5000 dilution) or HRP conjugated goat anti-mouse IgG (H+L) secondary antibody (Thermo Fisher Scientific, Cat. No. 62–6520, 1:5000 dilution) at room temperature for 1 h. All blots were developed with SuperSignal West Pico Chemiluminescence Substrate or SuperSignal West Femto Maximum Sensitivity Substrate (Thermo Fisher Scientific, Cat. No. 34095) with a ChemiDoc MP Imaging system.

#### cAMP assay

C6 glioma cells (10,000/well) were cultured in media in individual wells of a 96-well plate (Nunc, Cat. No. 165306) for 72 h. Glycosylation inhibitors were also added at the time of seeding to achieve these final concentrations: 100 μM NB-DGJ, 200 μM 2F-Fuc, and 200 μM 3Fax-NeuAc. Forskolin was purchased from Sigma-Aldrich (Cat. No. F6886); stock concentrations were made at 10 mM in DMSO. Cholera toxin (CT) or forskolin were diluted in DPBS supplemented with 500 μM IBMX (3-Isobutyl-1-methylxanthine; ACROS Organics, Cat. No. AC228420010; 100 mM stock in DMSO), 100 μM Ro 20–1724 (Tocris, Cat. No. 04-155-0; 100 mM stock in DMSO), and 25 mM MgCl_2_. Cells were washed once with room temperature DPBS, then either 40 μL of diluted CT was added per well and the plate incubated for 45 min at 37°C followed by 15 min at room temperature, or 40 μL of diluted forskolin was added per well and the plate incubated for 20 min at room temperature. For inhibition of retrograde transport, cells were pre-treated for 1 h at 37°C with 1 μg/mL brefeldin A (Sigma-Aldrich, Cat. No. B7651-5MG; stocks were 2.5 mg/mL in absolute ethanol), after which 1 μg/mL brefeldin A was included in the CT dilution buffer. Accumulated cAMP was measured using the cAMP-Glo Max assay (Promega, Cat. No. V1681) according to the manufacturer’s instructions. Luminescence was determined with a Synergy Neo microplate reader (BioTek).

## Supporting information

S1 Fig CTB binding to blood from ABO-donors and blocking of CTB binding to human and mouse leukocytes. **A)** Bar graphs showing gMFI of CTB binding to different cell types in human blood. n = 3–6 from each blood group (A, B, AB or O). **B)** Histogram and bar graph (n = 8) showing blocking of CTB binding to human monocytes by pre-treating CTB with sugars or pretreating the cells with lectins. **C)** Histogram and bar graph (n = 6) showing blocking of CTB binding to murine monocytes by pre-treating CTB with sugars or pretreating the cells with lectins. **D)** Bar graph (n = 5) showing blocking of CTB binding to human granulocytes by pretreating the cells with PNA. **E)** Representative (of 3 independent experiments) bar graph showing blocking of CTB binding to murine T cells by pretreating the cells with PNA or pretreating CTB with D-galactose. Significance was calculated using a one-way-ANOVA with Tukey correction (**** = p<0,0001, ** = p<0,01 and * = p<0,05).
(TIF)

S2 Fig Lectin and anti-Le^X^ binding to HL60 cells. **A)** Bar graphs from flow cytometry analysis of lectin binding to HL60 cells after treatment with glycosylation inhibitors. **B)** Flow cytometry analysis of anti-Le^X^ binding to undifferentiated HL-60 cells.
(TIF)

S3 Fig Lectin binding to os-HSA and blocking of CTB binding to Jurkat cells. **A)** ELISA with titrated amounts of os-linked to HSA, immobilized to wells, blocked with lectins prior to detection with CTB-HRP (top panel) or detected with biotin-linked lectins + streptavidin-HRP (bottom panel). **B)** Histogram and bar graph showing CTB binding to Jurkat cell line cells by either pre-treating the cells with lectins or pre-treating CTB with indicated os. The data are pooled from 5 independent experiments. Significance was calculated using a one-way-ANOVA with Tukey correction (**** = p<0,0001).
(TIF)

S4 Fig CTB Co-IP of T84 cells. Western blot using anti-Le^X^ of T84 cell lysate after incubation with CTB, lysis and immunoprecipitation with anti-CTB. One representative out of two independent experiments is shown.
(TIF)

S5 Fig CTB binding to jejunal epithelial cells from donors where all cells express Le^X^. **A)** Contour plot of CTB and anti-Le^X^ binding to EpCAM+ cells. **B)** CTB was pretreated or not with indicated sugars, os or os-HSA before used to stain cells or **C)** cells were pre-treated or not with lectins prior to staining with CTB, G33D or OVA. Graphs show the percent of gMFI of CTB binding to EpCAM+ cells where 100% represents CTB staining with no blocking. Data collected from a total of 7 donors and each dot represent measurements from one donor. **D)** Histogram of CTB binding to EpCAM+ cells after pretreating the cells with anti-Le^X^ antibody HI98. **E)** Bar graph showing CTB binding to EpCAM+ cells after pretreating the cells with anti-Le^X^ antibody HI98 in 3 donors (one shape represent the same donor).
(TIF)

S6 Fig Glycosylation of C6 cells and effects of glycosylation inhibitors. **A)** C6 cells were cultured with the indicated inhibitors for 72 h. After 20 min exposure to forskolin, accumulated cAMP was measured by the cAMP-Glo™ luminescence assay. Luminescence signal is inversely proportional to cAMP levels. **B)** Lysates from the indicated cell lines were separated by PAGE and probed with biotin-AAL, followed by streptavidin-peroxidase conjugate and development with chemiluminescent substrate. Equivalent amounts of protein were loaded in each lane. **C)** C6 cells were cultured with the indicated inhibitors for 72 h. Staining was performed with biotin-AAL, followed by DTAF-streptavidin. Fluorescence was measured by flow cytometry.
(TIF)

S7 Fig WB and HPLC-data on GSLs tissue from the murine small intestine. **A)** Bar graph showing concentration of all GSLs in middle section in murine small intestine of wt or KO mice. **B)** Bar graph showing the levels of GSLs present in the (proximal, middle or distal part of) murine small intestine for wt and KO. **C)** Bar graph showing concentration of all GSLs in middle section in murine small intestine of KO mice treated or not with NB-DNJ (n = 3–4). Error bars show SD. **D-E)** SDS-PAGE with subsequent western blot was performed on **(D)** sub-fractionated lysates or **(E)** whole lysates from murine small intestine. The membranes were probed with **(D)** CTB or **(E)** CTB with or without prior treatment with periodate (to selectively oxidize glycan modifications). **(F-G)** Histograms showing binding of **F)** anti-Le^X^ (clone HI98) or **G)** AAL-bio (with streptavidin-PE) to murine jejunal epithelial cells. FMO samples lack **F)** anti-Le^X^ or **G)** streptavidin-PE respectively.
(TIF)
