# Supplementary figures and images for: GM1 ganglioside-independent intoxication by Cholera toxin
Source: PLoS Pathog. 2018 Feb 12;14(2):e1006862. doi: 10.1371/journal.ppat.1006862 (PMC5825173; doi:10.1371/journal.ppat.1006862)

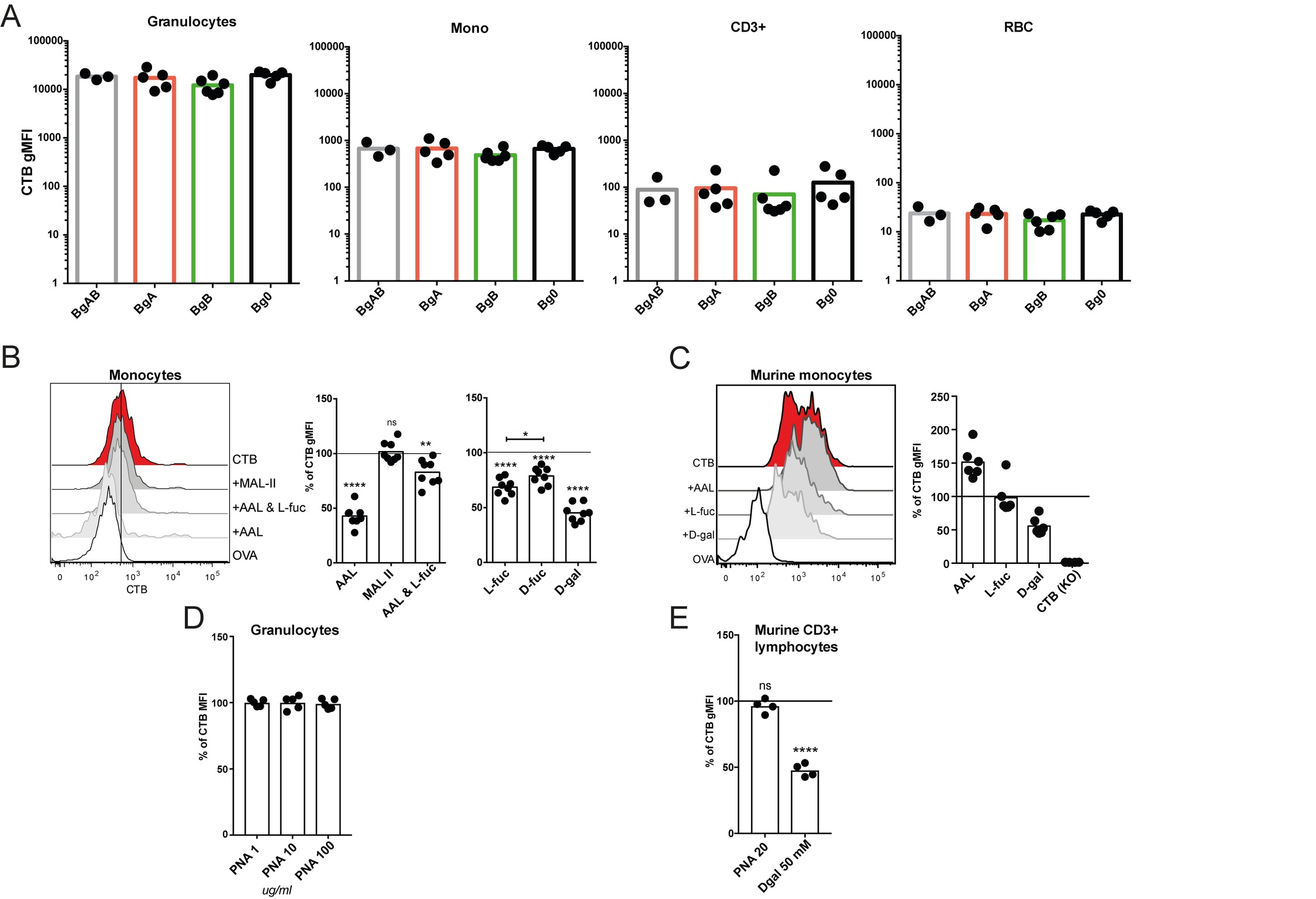

Supplement: S1 Fig — A) Bar graphs showing gMFI of CTB binding to different cell types in human blood. n = 3–6 from each blood group (A, B, AB or O). B) Histogram and bar graph (n = 8) showing blocking of CTB binding to human monocytes by pre-treating CTB with sugars or pretreating the cells with lectins. C) Histogram and bar graph (n = 6) showing blocking of CTB binding to murine monocytes by pre-treating CTB with sugars or pretreating the cells with lectins. D) Bar graph (n = 5) showing blocking of CTB binding to human granulocytes by pretreating the cells with PNA. E) Representative (of 3 independent experiments) bar graph showing blocking of CTB binding to murine T cells by pretreating the cells with PNA or pretreating CTB with D-galactose. Significance was calculated using a one-way-ANOVA with Tukey correction (**** = p<0,0001, ** = p<0,01 and * = p<0,05). (TIF) [file ppat.1006862.s001.tif]

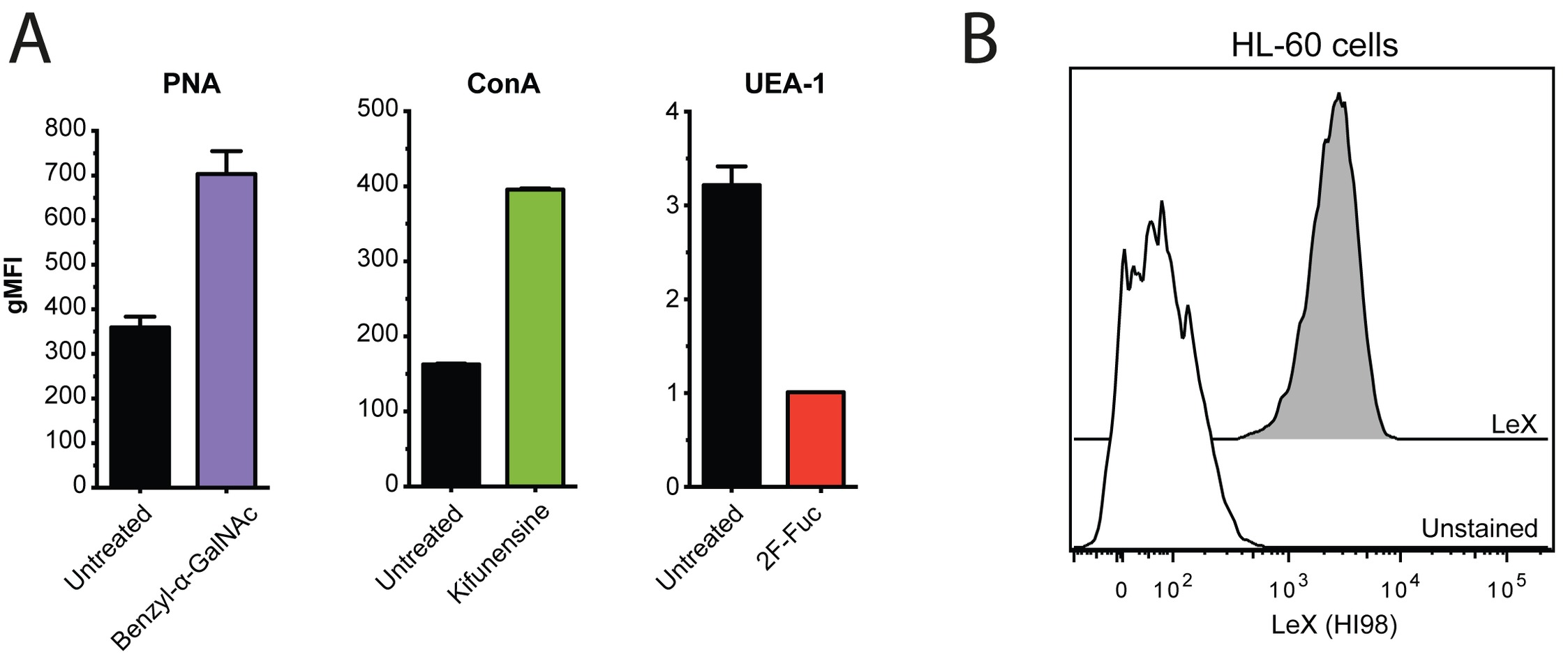

Supplement: S2 Fig — A) Bar graphs from flow cytometry analysis of lectin binding to HL60 cells after treatment with glycosylation inhibitors. B) Flow cytometry analysis of anti-LeX binding to undifferentiated HL-60 cells. (TIF) [file ppat.1006862.s002.tif]

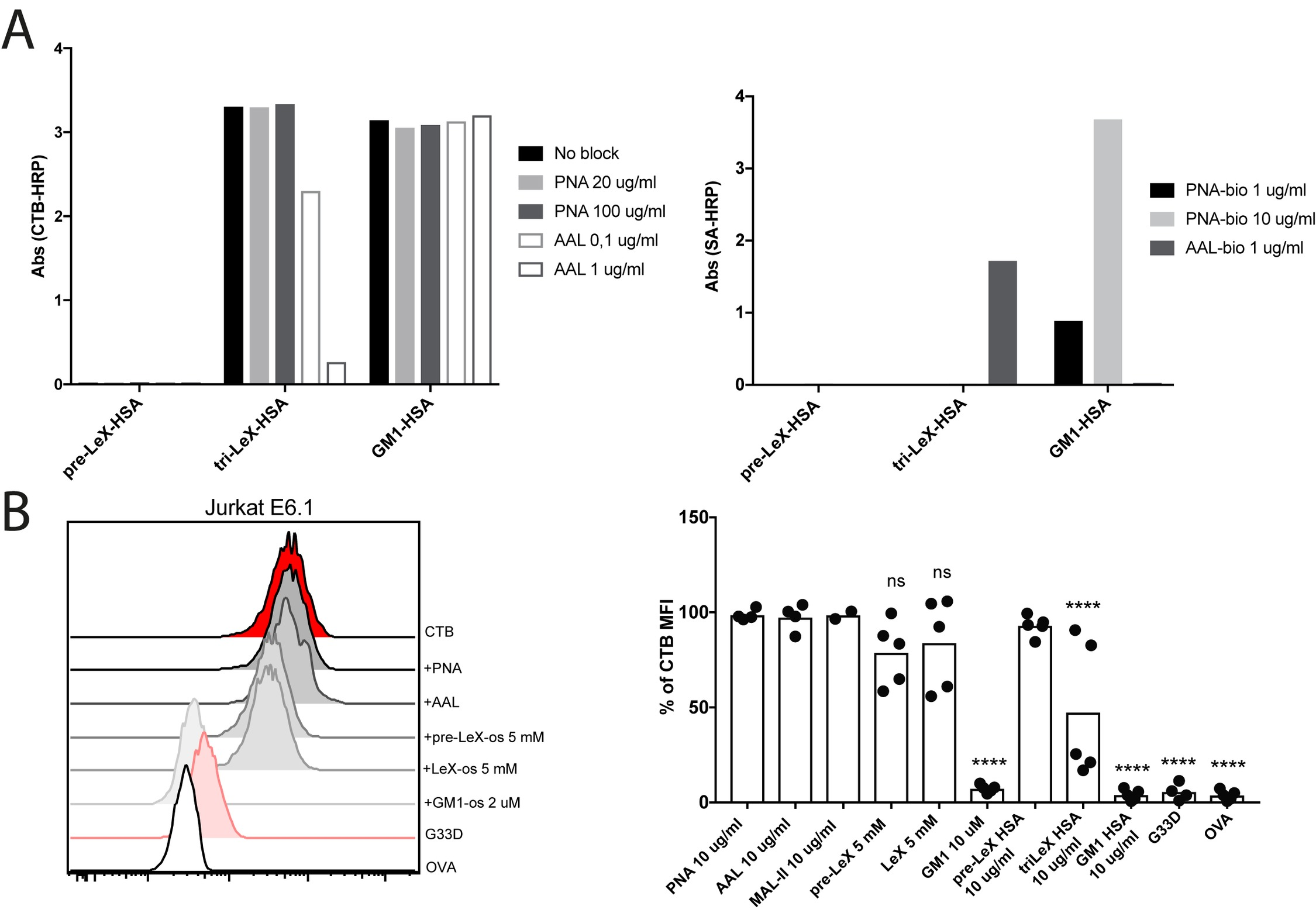

Supplement: S3 Fig — A) ELISA with titrated amounts of os-linked to HSA, immobilized to wells, blocked with lectins prior to detection with CTB-HRP (top panel) or detected with biotin-linked lectins + streptavidin-HRP (bottom panel). B) Histogram and bar graph showing CTB binding to Jurkat cell line cells by either pre-treating the cells with lectins or pre-treating CTB with indicated os. The data are pooled from 5 independent experiments. Significance was calculated using a one-way-ANOVA with Tukey correction (**** = p<0,0001). (TIF) [file ppat.1006862.s003.tif]

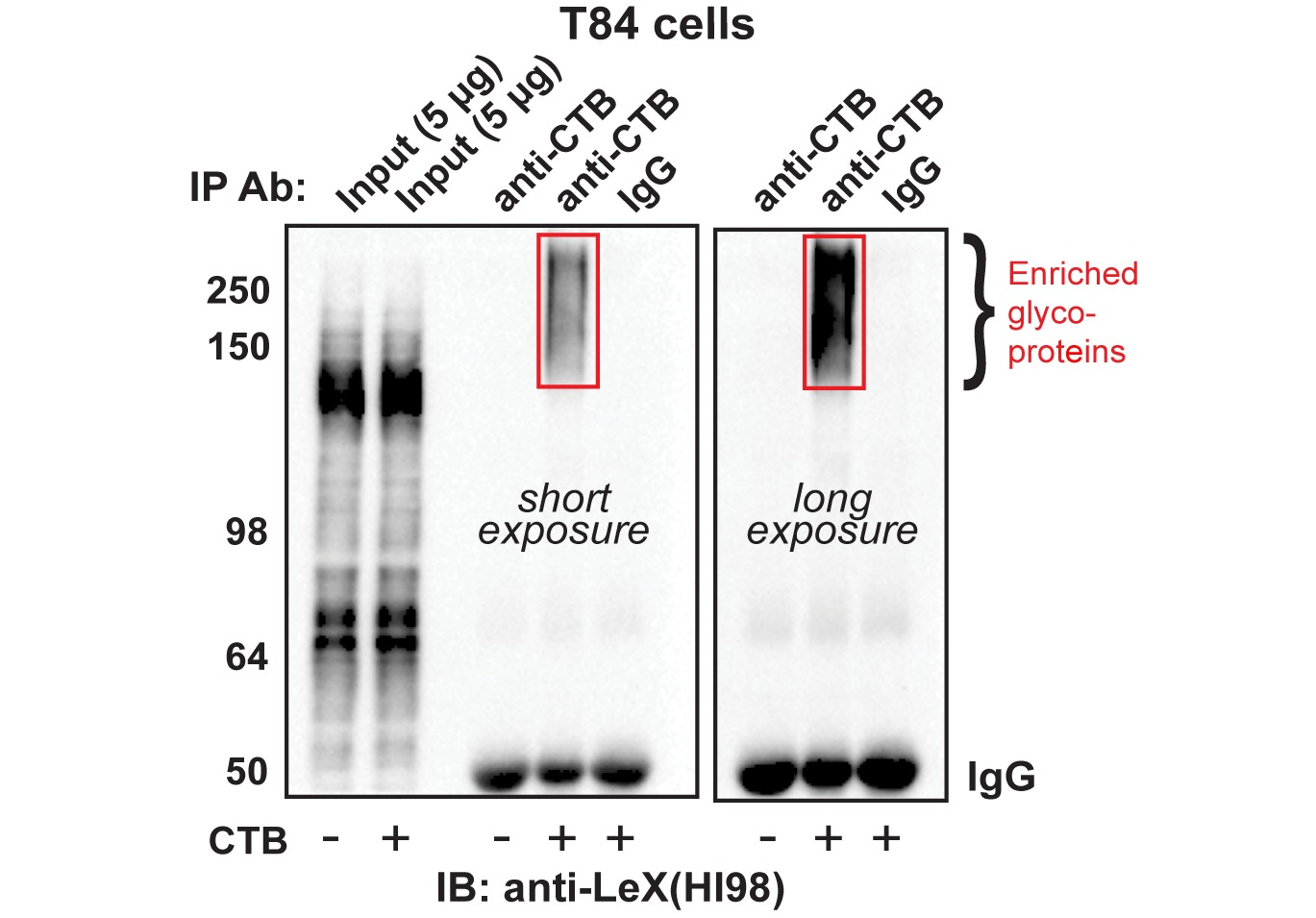

Supplement: S4 Fig — Western blot using anti-LeX of T84 cell lysate after incubation with CTB, lysis and immunoprecipitation with anti-CTB. One representative out of two independent experiments is shown. (TIF) [file ppat.1006862.s004.tif]

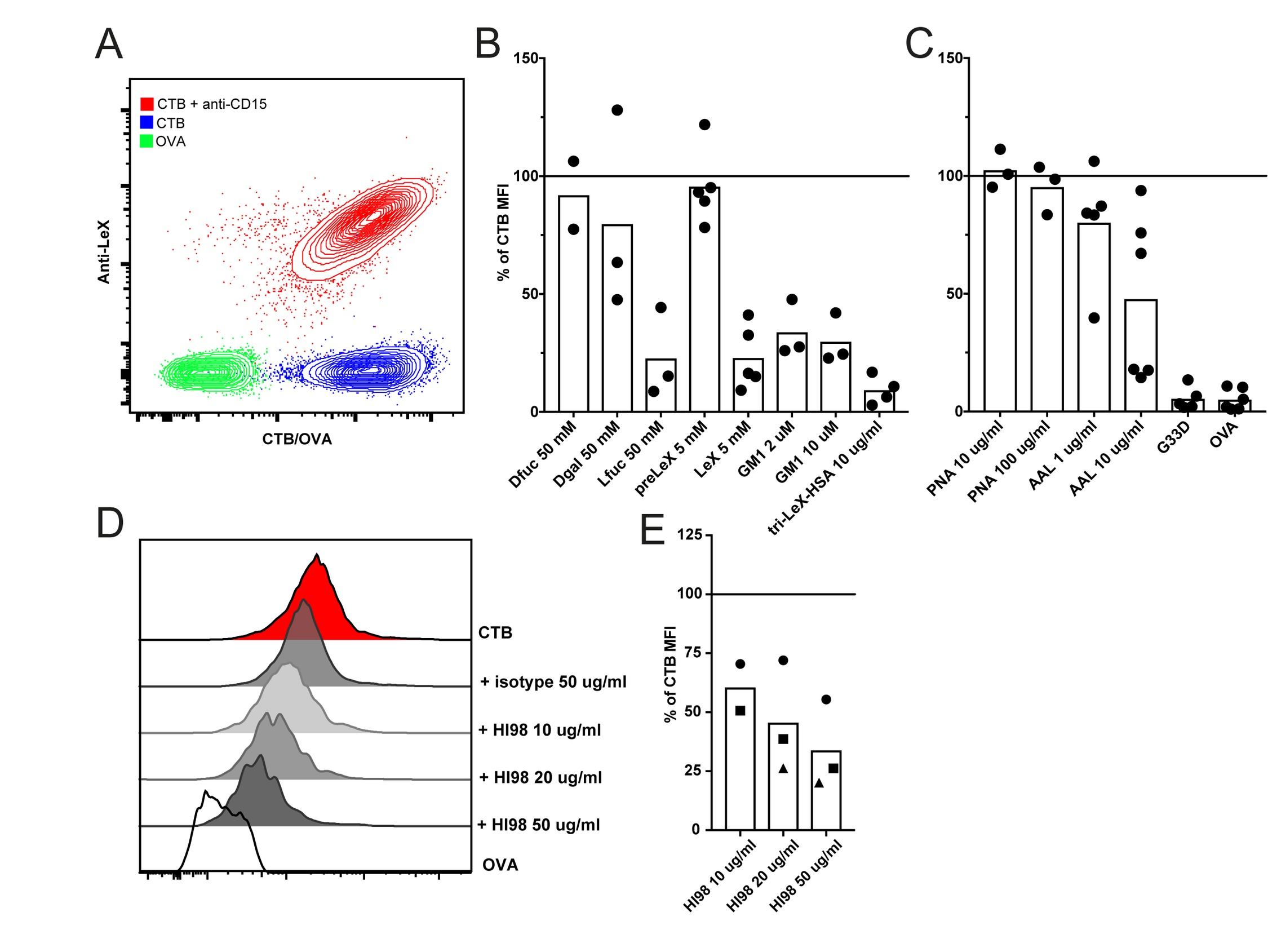

Supplement: S5 Fig — A) Contour plot of CTB and anti-LeX binding to EpCAM+ cells. B) CTB was pretreated or not with indicated sugars, os or os-HSA before used to stain cells or C) cells were pre-treated or not with lectins prior to staining with CTB, G33D or OVA. Graphs show the percent of gMFI of CTB binding to EpCAM+ cells where 100% represents CTB staining with no blocking. Data collected from a total of 7 donors and each dot represent measurements from one donor. D) Histogram of CTB binding to EpCAM+ cells after pretreating the cells with anti-LeX antibody HI98. E) Bar graph showing CTB binding to EpCAM+ cells after pretreating the cells with anti-LeX antibody HI98 in 3 donors (one shape represent the same donor). (TIF) [file ppat.1006862.s005.tif]

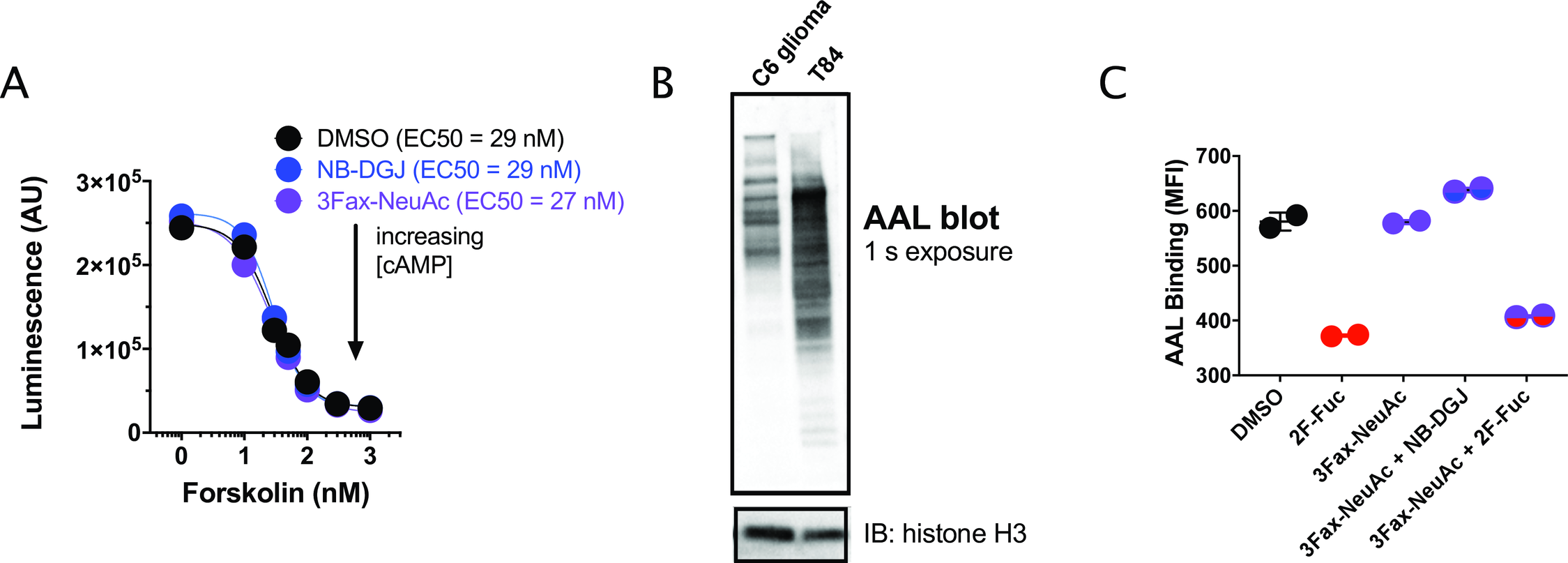

Supplement: S6 Fig — A) C6 cells were cultured with the indicated inhibitors for 72 h. After 20 min exposure to forskolin, accumulated cAMP was measured by the cAMP-Glo™ luminescence assay. Luminescence signal is inversely proportional to cAMP levels. B) Lysates from the indicated cell lines were separated by PAGE and probed with biotin-AAL, followed by streptavidin-peroxidase conjugate and development with chemiluminescent substrate. Equivalent amounts of protein were loaded in each lane. C) C6 cells were cultured with the indicated inhibitors for 72 h. Staining was performed with biotin-AAL, followed by DTAF-streptavidin. Fluorescence was measured by flow cytometry. (TIF) [file ppat.1006862.s006.tif]

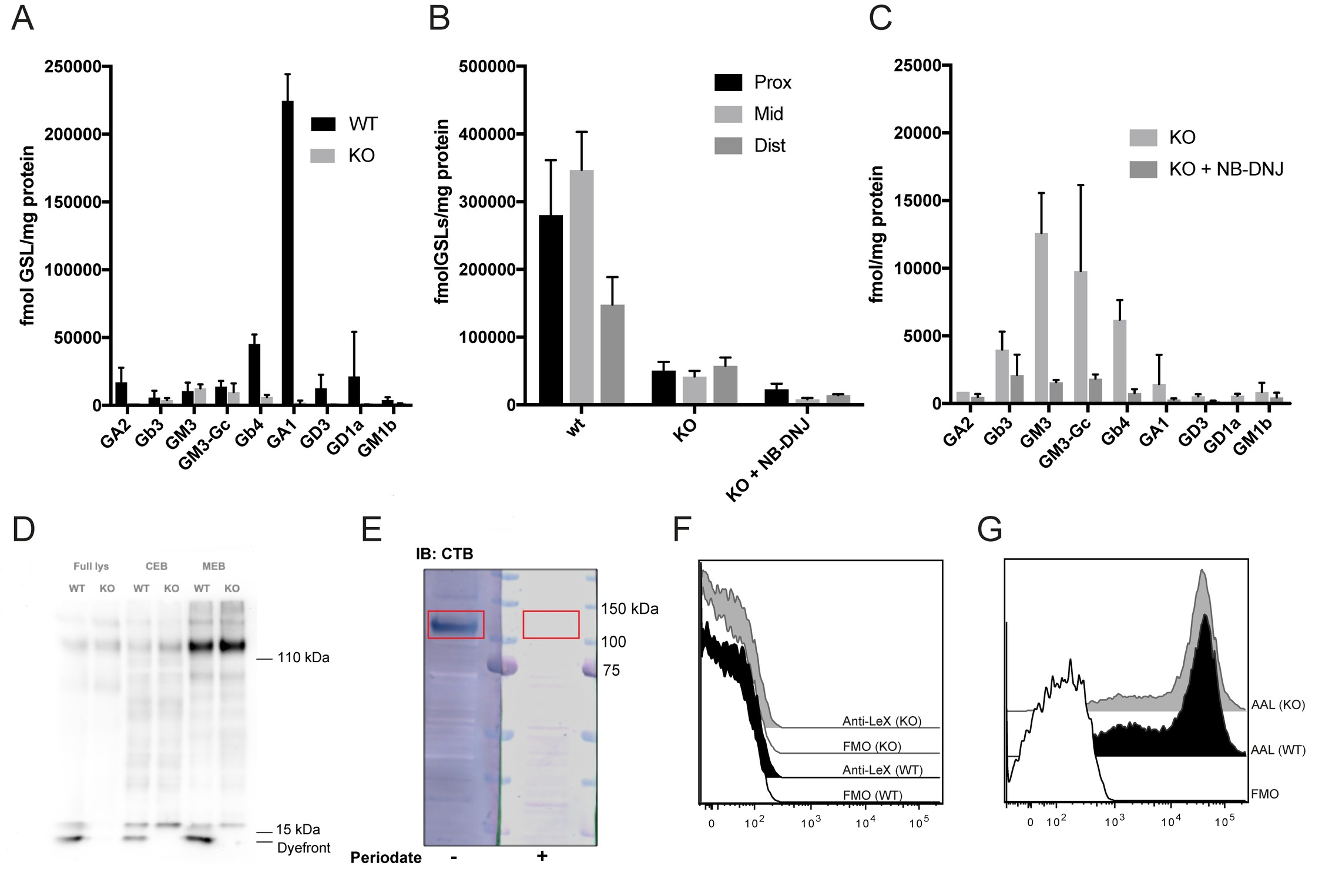

Supplement: S7 Fig — A) Bar graph showing concentration of all GSLs in middle section in murine small intestine of wt or KO mice. B) Bar graph showing the levels of GSLs present in the (proximal, middle or distal part of) murine small intestine for wt and KO. C) Bar graph showing concentration of all GSLs in middle section in murine small intestine of KO mice treated or not with NB-DNJ (n = 3–4). Error bars show SD. D-E) SDS-PAGE with subsequent western blot was performed on (D) sub-fractionated lysates or (E) whole lysates from murine small intestine. The membranes were probed with (D) CTB or (E) CTB with or without prior treatment with periodate (to selectively oxidize glycan modifications). (F-G) Histograms showing binding of F) anti-LeX (clone HI98) or G) AAL-bio (with streptavidin-PE) to murine jejunal epithelial cells. FMO samples lack F) anti-LeX or G) streptavidin-PE respectively. (TIF) [file ppat.1006862.s007.tif]
